# Supplementary material for: Understanding the interplay of GDP, renewable, and non-renewable energy on carbon emissions: Global wavelet coherence and Granger causality analysis
Source: PLoS One. 2024 Sep 19;19(9):e0308780. doi: 10.1371/journal.pone.0308780 (PMC11412634; doi:10.1371/journal.pone.0308780)
Supplement: S2 Appendix — (DOCX) [file pone.0308780.s002.docx]

**S2 Appendix: Cross-country analysis between CO_2_ and PGDP for all income categories using Granger Causality**

| **High-Income Country Category** | | | | | |
| --- | --- | --- | --- | --- | --- |
| **Country** | **DCO_2_→DGDP** | **DGDP →DCO_2_** | **Country** | **DCO_2_→DGDP** | **DGDP → DCO_2_** |
| Finland | 7.4549*** | 8.4473*** | Andorra | 0.17831 | 1.9948 |
| Poland | 7.1726** | 9.4147*** | Australia | 0.80224 | 1.2234 |
| Romania | 4.1022** | 4.6068** | Belgium | 1.7598 | 0.56036 |
| Slovak Republic | 4.3606** | 3.8855** | Denmark | 0.30938 | 0.65297 |
| Austria | 0.10395 | 5.1172** | France | 2.4799 | 4.3853 |
| Barbados | 11.4*** | 0.07047 | Iceland | 0.81819 | 0.11364 |
| Canada | 0.90819 | 7.7598** | Ireland | 0.74743 | 1.9462 |
| Chile | 5.3632* | 0.15808 | Israel | 1.7977 | 0.41355 |
| Cyprus | 4.8958** | 3.393 | Italy | 1.0262 | 3.4285 |
| Czechia | 0.53789 | 6.906** | Liechtenstein | 1.1093 | 1.2296 |
| Germany | 9.0659** | 4.3317 | Norway | 1.6676 | 1.4144 |
| Greece | 5.7334* | 0.24463 | Portugal | 4.4992 | 1.054 |
| Guyana | 4.9038* | 2.2539 | Qatar | 0.67629 | 0.20489 |
| Hungary | 8.6624** | 2.693 | Switzerland | 2.3255 | 0.07649 |
| Japan | 2.3704 | 15.379*** | United Arab Emirates | 0.16574 | 2.6976 |
| Korea, Rep. | 3.2617* | 0.22096 | United Kingdom | 0.36725 | 7.2247 |
| Netherlands | 3.189 | 4.893* | United States | 0.53033 | 0.16375 |
| New Zealand | 8.3178** | 2.6661 |  | **DCO_2_→DDGDP** | **DDGDP→DCO_2_** |
| Saudi Arabia | 3.6512* | 2.1274 | Seychelles | 3.0623 | 1.2442 |
| Singapore | 5.1513* | 1.1697 | St. Kitts and Nevis | 0.15521 | 0.38054 |
| Spain | 8.7631** | 3.7188 | Uruguay | 0.00037 | 0.12369 |
| Sweden | 9.4446*** | 0.98366 |  | **DDCO_2_ → DGDP** | **DGDP → DDCO_2_** |
| Trinidad and Tobago | 4.0436 | 18.845*** | Luxembourg | 1.6318 | 4.4743 |
|  |  |  |  | **DCO_2_→DDDDGDP** | **DDDDGDP→CO_2_** |
|  |  |  | Panama | 0.74596 | 0.92191 |

| **Low-Income Country Category** | | | | | |
| --- | --- | --- | --- | --- | --- |
| **Country** | **CO_2_ → DGDP** | **DGDP → CO_2_** | **Country** | **DCO_2_→DGDP** | **DGDP → DCO_2_** |
| Togo | 10.892*** | 0.89598 | Burundi | 0.5031 | 0.91717 |
|  | **DCO_2_→DGDP** | **DGDP →DCO_2_** | Congo,Dem.Rep. | 0.36398 | 1.6154 |
| Central African Republic | 10.956*** | 0.12232 | Ethiopia | 1.0345 | 3.9081 |
| Chad | 6.0916** | 1.517 | Gambia, The | 0.47744 | 2.9759 |
| Madagascar | 0.0756 | 6.575** | Guinea-Bissau | 1.4624 | 0.27401 |
| Mali | 0.06785 | 27.092*** | Malawi | 1.2926 | 1.1866 |
| Niger | 12.255*** | 0.06262 | Mozambique | 2.4077 | 1.5283 |
| Sudan | 0.000072 | 6.3733** | Rwanda | 0.16437 | 3.628 |
| Syrian Arab Republic | 6.4452** | 0.20852 | Sierra Leone | 1.9934 | 1.709 |
| Uganda | 0.59245 | 12.1*** |  | **DCO_2_→DDGDP** | **DDGDP →DCO_2_** |
| Burkina Faso | 1.9299 | 4.345 | Yemen, Rep. | 0.65074 | 1.0079 |

| **Upper Middle Income Country Category** | | | | | | |
| --- | --- | --- | --- | --- | --- | --- |
| **Country** | **CO_2_ → DGDP** | **DGDP → CO_2_** | **Country** | | **DCO_2_→DGDP** | **DGDP → DCO_2_** |
| Dominican Republic | 4.0343** | 3.905** | Georgia | | 1.1348 | 2.6958 |
| Azerbaijan | 0.00855 | 2.3087 | Grenada | | 1.1168 | 2.9416 |
| Cuba | 0.08223 | 0.03591 | Guatemala | | 0.10125 | 0.13765 |
| Fiji | 1.4319 | 2.9293 | Indonesia | | 3.4144 | 1.3449 |
| Tonga | 0.2379 | 4.2178 | Iraq | | 0.47799 | 1.3124 |
|  | **CO_2_→DDGDP** | **DDGDP →CO_2_** | Kazakhstan | | 0.9042 | 4.8706 |
| Libya | 0.00318 | 13.318*** | Mauritius | | 2.034 | 0.79698 |
| Belize | 1.9404 | 3.2082 | Namibia | | 3.4006 | 3.0756 |
|  | **DCO_2_→DGDP** | **DGDP →DCO_2_** | North Macedonia | | 3.9921 | 1.8184 |
| Albania | 6.0174** | 1.0094 | Paraguay | | 0.8768 | 1.109 |
| Botswana | 0.63726 | 4.9977* | Peru | | 2.2034 | 0.00102 |
| Dominica | 6.0176** | 1.0094 | Russian Federation | | 0.3082 | 0.77589 |
| El Salvador | 4.6085 | 5.482* | South Africa | | 1.6203 | 3.4487 |
| Gabon | 2.5442 | 5.8213* | Thailand | 1.4933 | | 1.7763 |
| Jamaica | 1.3444 | 6.9757** | Turkiye | 0.48031 | | 0.23269 |
| Malaysia | 0.10041 | 4.6138** | Turkmenistan | 0.82147 | | 4.2335 |
| Mexico | 0.60621 | 10.786*** |  | **DDCO_2_→DGDP** | | **DGDP →DDCO_2_** |
| Argentina | 0.88552 | 4.5925 | Marshall Islands | 5.3365** | | 0.1256 |
| Armenia | 2.0725 | 0.10074 |  | **DCO_2_→DDGDP** | | **DDGDP →DCO_2_** |
| Belarus | 3.0539 | 3.4499 | St. Vincent and the Grenadines | 2.7765* | | 0.01772 |
| Brazil | 2.6769 | 0.20026 | Maldives | 2.5324 | | 0.08755 |
| Bulgaria | 2.4648 | 1.5033 | St. Lucia | 1.0916 | | 0.02027 |
| Colombia | 1.9556 | 1.0097 |  | **DDCO_2_→DDGDP** | | **DDGDP→DDCO_2_** |
| Costa Rica | 2.9688 | 1.5815 | China | 2.8607 | | 2.9558 |
| Ecuador | 0.1932 | 1.8087 |  |  | |  |
| Equatorial Guinea | 0.14715 | 3.5986 |  |  | |  |

| **Lower Middle Income Country Category** | | | | | |
| --- | --- | --- | --- | --- | --- |
| **Country** | **DCO_2_→DGDP** | **DGDP →DCO_2_** | **Country** | **DCO_2_→DGDP** | **DGDP → DCO_2_** |
| Algeria | 8.7033** | 1.824 | Myanmar | 1.295 | 2.0621 |
| Angola | 9.0286*** | 1.5147 | Nigeria | 3.3642 | 1.0683 |
| Benin | 7.8537** | 2.4087 | Pakistan | 1.003 | 1.2693 |
| Cabo Verde | 0.94622 | 5.9094* | Papua New Guinea | 3.0883 | 2.2601 |
| Ghana | 1.9587 | 4.3895** | Philippines | 0.91436 | 2.4858 |
| Guinea | 0.70189 | 3.2148* | Senegal | 0.10778 | 0.69372 |
| Haiti | 6.3848** | 0.67155 | Solomon Islands | 2.8097 | 1.4925 |
| Honduras | 3.2372 | 5.2295* | Sri Lanka | 1.6694 | 0.58589 |
| Kiribati | 8.4189*** | 0.30607 | Tanzania | 1.1154 | 1.3665 |
| Lesotho | 7.7475** | 0.2494 | Ukraine | 0.91533 | 0.98501 |
| Mongolia | 5.5642* | 3.7729 |  | **DCO_2_→DDGDP** | **DDGDP →DCO_2_** |
| Nepal | 0.00878 | 13.808*** | Uzbekistan | 2.668 | 1.7081 |
| Samoa | 3.9022** | 0.00532 | Viet Nam | 1.2865 | 0.22948 |
| Tajikistan | 4.7394** | 0.94469 | Zambia | 3.0667 | 1.2792 |
| Tunisia | 5.158* | 0.01338 | Zimbabwe | 1.3214 | 0.61747 |
| Cameroon | 0.1553 | 2.2843 |  | **CO_2_ → DGDP** | **DGDP → CO_2_** |
| Comoros | 0.43594 | 0.80252 | Vanuatu | 0.24639 | 5.1787* |
| Cote d'Ivoire | 0.68191 | 0.15652 | Congo, Rep. | 0.78679 | 0.13529 |
| Djibouti | 26.573 | 0.0582 | Eswatini | 0.25849 | 2.0183 |
| Egypt, Arab Rep. | 3.7374 | 1.053 | Nicaragua | 0.0675 | 0.01865 |
| India | 0.78251 | 0.7669 |  | **DDCO_2_ → DGDP** | **DGDP →DDCO_2_** |
| Iran, Islamic Rep. | 2.3871 | 0.25252 | Bangladesh | 2.8759* | 0.07605 |
| Jordan | 1.557 | 3.2651 | Bhutan | 0.82739 | 4.9009* |
| Kenya | 3.602 | 0.86505 | Lao PDR | 2.5967 | 1.2587 |
| Kyrgyz Republic | 6.1369 | 4.6636 |  | **DDCO_2_→DDGDP** | **DDGDP→DDCO_2_** |
| Mauritania | 1.7135 | 1.4027 | Bolivia | 0.88439 | 2.2497 |
| Micronesia, Fed.Sts. | 0.09323 | 0.5259 |  | **DCO_2_ → DDDGDP** | **DDDDGDP→CO_2_** |
| Morocco | 1.4402 | 2.2672 | Lebanon | 2.1861 | 3.563 |

Note: The symbols *, **, *** depicts 10%, 5%, 1% significance level respectively.

Source: Author’s Compilation
