# Supplementary material for: Understanding the interplay of GDP, renewable, and non-renewable energy on carbon emissions: Global wavelet coherence and Granger causality analysis
Source: PLoS One. 2024 Sep 19;19(9):e0308780. doi: 10.1371/journal.pone.0308780 (PMC11412634; doi:10.1371/journal.pone.0308780)
Supplement: S3 Appendix — (DOCX) [file pone.0308780.s003.docx]

**S3 Appendix: Cross-country analysis between CO_2_ and RE for all income categories using Granger Causality**

| **High-Income Country Category** | | | | | |
| --- | --- | --- | --- | --- | --- |
| **Country** | **DCO_2_→RE** | **RE → DCO_2_** | **Country** | **DCO_2_→DRE** | **DRE → DCO_2_** |
| Qatar | 4.8659** | 3.5911* | Chile | 2.773 | 1.8663 |
| Trinidad and Tobago | 12.332*** | 4.6924*** | Czechia | 2.3305 | 1.7932 |
| Seychelles | 4.6034** | 0.15247 | Denmark | 0.76338 | 2.8243 |
|  | **DCO_2_→DRE** | **DRE → DCO_2_** | Greece | 1.5475 | 0.90249 |
| France | 16.672*** | 5.3093* | Guyana | 3.2565 | 1.2851 |
| Portugal | 5.4013* | 7.79** | Hungary | 1.2216 | 1.9441 |
| Romania | 6.419** | 25.856*** | Ireland | 0.70788 | 3.6151 |
| Uruguay | 7.1372** | 11.022*** | Italy | 0.01679 | 1.872 |
| Andorra | 1.3578 | 7.3215** | Japan | 1.9166 | 0.42093 |
| Australia | 4.2126** | 1.4478 | Korea, Rep. | 3.1094 | 0.20903 |
| Canada | 1.6367 | 5.1549* | Liechtenstein | 2.1271 | 3.1828 |
| Cyprus | 6.4384** | 0.22936 | Norway | 0.39451 | 2.4081 |
| Finland | 6.4582** | 0.28838 | Poland | 2.1932 | 2.559 |
| Germany | 7.0183** | 0.1037 | Slovak Republic | 0.37797 | 2.3573 |
| Iceland | 0.92878 | 8.2621** | Sweden | 0.34543 | 0.77822 |
| Israel | 18.592*** | 2.7572 | United Arab Emirates | 1.5249 | 1.3539 |
| New Zealand | 6.5781** | 1.7915 | United States | 0.01754 | 2.449 |
| Panama | 1.2449 | 12.54*** |  | **DDCO_2_→DRE** | **DRE → DDCO_2_** |
| Singapore | 2.0825 | 20.079*** | Luxembourg | 1.0157 | 2.5853 |
| Spain | 1.9144 | 10.701*** |  | **DCO_2_→DDRE** | **DDRE → DCO_2_** |
| St. Kitts and Nevis | 3.3179 | 20.156*** | Saudi Arabia | 0.01485 | 15.004*** |
| Switzerland | 0.11535 | 3.2718* | United Kingdom | 0.32719 | 1.9686 |
| Austria | 3.1073 | 2.6879 |  | **DCO_2_ → DDRE** | **DDRE → DCO_2_** |
| Barbados | 0.23425 | 0.69544 | Netherlands | 0.20705 | 0.64123 |
| Belgium | 2.0842 | 0.77784 |  |  |  |

| **Low-Income Country Category** | | | | | |
| --- | --- | --- | --- | --- | --- |
| **Country** | **CO_2_ → RE** | **RE → CO_2_** | **Country** | **DCO_2_→DRE** | **DRE → DCO_2_** |
| Togo | 1.2603 | 0.99064 | Syrian Arab Republic | 1.0458 | 5.2023* |
|  | **DCO_2_→RE** | **RE → DCO_2_** | Burkina Faso | 0.00346 | 0.90512 |
| Yemen, Rep. | 0.38328 | 0.70389 | Burundi | 1.4724 | 2.5628 |
|  | **DCO_2_→DRE** | **DRE → DCO_2_** | Ethiopia | 2.7615 | 2.7289 |
| Central African Republic | 21.379*** | 30.628*** | Gambia, The | 0.75818 | 0.2634 |
| Chad | 9.0351*** | 14.128*** | Madagascar | 0.3891 | 0.83152 |
| Congo, Dem. Rep. | 12.771*** | 11.193*** | Malawi | 0.03557 | 0.23168 |
| Guinea-Bissau | 4.4611 | 11.01*** | Mozambique | 0.73739 | 0.45663 |
| Mali | 5.2105* | 1.2816 | Sierra Leone | 0.65604 | 1.8036 |
| Niger | 3.8535 | 8.8655** |  | **DCO_2_→DDRE** | **DDRE → DCO_2_** |
| Rwanda | 9.6032** | 0.42936 | Uganda | 5.2234* | 2.4525 |
| Sudan | 1.734 | 6.81** |  |  |  |

| **Upper Middle Income Country Category** | | | | | |
| --- | --- | --- | --- | --- | --- |
| **Country** | **CO_2_ → RE** | **RE → CO_2_** | **Country** | **DCO_2_→DRE** | **DRE → DCO_2_** |
| Dominican Republic | 0.1774 | 0.21958 | Armenia | 0.00759 | 0.66358 |
|  | **CO_2_ → DRE** | **DRE → CO_2_** | Brazil | 0.28482 | 2.9404 |
| Belize | 8.9573*** | 0.10644 | Bulgaria | 0.41412 | 3.668 |
| Cuba | 1.9631 | 11.054*** | Dominica | 1.623 | 1.0407 |
| Fiji | 0.00229 | 4.4649** | Ecuador | 1.7235 | 3.0961 |
| Azerbaijan | 2.5628 | 0.4271 | El Salvador | 0.56828 | 0.96878 |
| Libya | 0.32741 | 1.3901 | Gabon | 0.6535 | 0.07253 |
| Tonga | 1.0079 | 2.0763 | Georgia | 1.696 | 2.1302 |
|  | **DCO_2_→RE** | **RE → DCO_2_** | Grenada | 1.0436 | 2.8862 |
| St. Lucia | 5.8153* | 4.8196* | Iraq | 1.4828 | 0.47018 |
| Guatemala | 2.9478* | 0.01113 | Malaysia | 3.1504 | 0.14179 |
| Mauritius | 4.4444** | 0.07451 | Mexico | 1.7346 | 1.7868 |
| Belarus | 0.76148 | 0.84314 | Namibia | 3.6519 | 0.93874 |
| Botswana | 0.53011 | 0.04306 | North Macedonia | 0.88219 | 0.00043 |
| Colombia | 0.80449 | 0.50625 | Paraguay | 0.00854 | 0.71535 |
| Russian Federation | 0.00806 | 2.5247 | Peru | 0.12568 | 1.097 |
|  | **DCO_2_→DRE** | **DRE → DCO_2_** | St. Vincent and the Grenadines | 0.09745 | 0.07382 |
| Equatorial Guinea | 3.0215* | 24.73*** | Thailand | 0.28967 | 0.04087 |
| Maldives | 4.2992** | 8.9082*** | Turkmenistan | 3.2861 | 2.4694 |
| Costa Rica | 0.92724 | 4.1204** |  | **DDCO_2_→DRE** | **DRE → DDCO_2_** |
| Indonesia | 2.7066 | 7.0836** | Marshall Islands | 0.19031 | 1.3771 |
| Jamaica | 4.9447** | 0.4193 |  | **DDCO_2_→DDRE** | **DDRE → DDCO_2_** |
| Kazakhstan | 2.2826 | 5.849** | China | 1.2331 | 0.19735 |
| Turkiye | 4.8182* | 0.21199 |  | **DCO_2_→DDRE** | **DDRE → DCO_2_** |
| Albania | 1.6229 | 1.0406 | South Africa | 16.52*** | 0.41366 |
| Argentina | 0.43015 | 1.0849 |  |  |  |

| **Lower Middle Income Country Category** | | | | | | |
| --- | --- | --- | --- | --- | --- | --- |
| **Country** | **CO_2_ → RE** | **RE → CO_2_** | **Country** | **DCO_2_→DRE** | | **DRE → DCO_2_** |
| Nicaragua | 0.17673 | 0.59555 | Lesotho | 3.9289 | | 1.0103 |
|  | **CO_2_ → DRE** | **DRE → CO_2_** | Mauritania | 1.7885 | | 2.4129 |
| Congo, Rep. | 1.7373 | 4.4092 | Micronesia, Fed. Sts. | 0.26908 | | 0.64291 |
| Eswatini | 0.31485 | 0.10665 | Morocco | 1.7476 | | 2.2051 |
| Vanuatu | 0.02976 | 2.536 | Myanmar | 1.8061 | | 1.2044 |
|  | **DCO_2_→RE** | **RE → DCO_2_** | Nepal | 0.61541 | | 0.33193 |
| Benin | 0.41215 | 0.02738 | Nigeria | 2.6214 | | 0.64313 |
| Kyrgyz Republic | 0.42392 | 0.05759 | Pakistan | 0.42863 | | 0.05116 |
| Uzbekistan | 1.4404 | 1.8246 | Papua New Guinea | | 1.2756 | 0.30513 |
|  |  |  | Philippines | | 3.2687 | 3.0764 |
| Guinea | 4.4475** | 5.9646** | Samoa | | 0.33894 | 0.76888 |
| Algeria | 1.3767 | 7.4413** | Senegal | | 0.00991 | 0.3816 |
| Cabo Verde | 0.52208 | 8.0724** | Solomon Islands | | 0.00519 | 0.06089 |
| Egypt, Arab Rep. | 0.13113 | 12.389*** | Sri Lanka | | 2.1168 | 2.9247 |
| Honduras | 4.6964* | 1.9579 | Tajikistan | | 2.2901 | 1.0912 |
| India | 2.8611* | 0.06135 | Tanzania | | 0.1339 | 0.74495 |
| Mongolia | 0.32254 | 8.045*** | Viet Nam | | 6.468 | 1.2071 |
| Tunisia | 6.5599* | 0.06265 | Zambia | | 1.3887 | 0.17693 |
| Angola | 2.5463 | 2.3308 | Zimbabwe | | 2.1289 | 1.7717 |
| Cameroon | 0.27601 | 2.161 |  | | **DDCO_2_→DRE** | **DRE → DDCO_2_** |
| Comoros | 1.9442 | 0.49203 | Bangladesh | | 0.78411 | 0.00231 |
| Cote d'Ivoire | 1.5464 | 4.371 | Lao PDR | | 0.92277 | 1.2922 |
| Djibouti | 0.95899 | 2.4652 |  | | **DDCO_2_→DDRE** | **DDRE → DDCO_2_** |
| Ghana | 0.53404 | 0.02525 | Bolivia | | 0.0002 | 2.7975* |
| Haiti | 0.35731 | 3.0392 | Bhutan | | 0.58184 | 1.4791 |
| Iran, Islamic Rep. | 0.58546 | 0.16672 |  | | **DCO_2_→DDRE** | **DDRE → DCO_2_** |
| Kenya | 2.7545 | 3.1165 | Jordan | | 0.72668 | 1.2044 |
| Kiribati | 1.5074 | 0.68635 | Ukraine | | 2.518 | 0.10261 |
| Lebanon | 1.078 | 3.1966 |  | |  |  |

Note: The symbols *, **, *** depicts 10%, 5%, 1% significance level respectively.

Source: Author’s Compilation
