# Supplementary material for: Understanding the interplay of GDP, renewable, and non-renewable energy on carbon emissions: Global wavelet coherence and Granger causality analysis
Source: PLoS One. 2024 Sep 19;19(9):e0308780. doi: 10.1371/journal.pone.0308780 (PMC11412634; doi:10.1371/journal.pone.0308780)
Supplement: Attachment — Submitted filename: Response to Reviewers.pdf [file pone.0308780.s006.pdf]

Point-by-Point Response to the Academic Editor and Reviewers Comments

Dear Editor and Reviewers,

We extend our sincere appreciation for the invaluable comments provided during the initial revision round, which greatly contributed to improving the paper to its current stage. We are grateful for your dedicated time and effort, as well as the insightful feedback from the reviewers, all of which have significantly enhanced the quality of our manuscript.

We are genuinely grateful for the opportunity to submit the second revised version of our manuscript, titled "Understanding the Interplay of GDP, Renewable, and Non-Renewable Energy on Carbon Emissions: Global Wavelet Coherence and Granger Causality Analysis" to PLOS ONE, initially submitted on 31st March 2024. We have diligently incorporated the suggested improvements, and the revised document includes clearly marked modifications.

As suggested, we have prepared a table detailing the changes, with line numbers corresponding to the revised manuscript that incorporates without track changes as follows. Your continued support and guidance are highly valued, and we look forward to any further insights you or the reviewers may offer.

Please find the reference table below, aligning with the line numbers in the revised manuscript containing without track changes.

**Journal Requirements**	
Requirement #1	***Response by Authors to Requirement #1***
Please review your reference list to ensure that it is complete and correct. If you have cited papers that have been retracted, please include the rationale for doing so in the manuscript text, or remove these references and replace them with relevant current references. Any changes to the reference list should be mentioned in the rebuttal letter that accompanies your revised manuscript. If you need to cite a retracted article, indicate the article's retracted status in the References list and also include a citation and full reference for the retraction notice.	We sincerely appreciate your careful review and valuable feedback on the reference list.<br><br>We have thoroughly reviewed our citations and made necessary revisions. Upon rechecking our reference list, we identified that reference ("Zhang et al., 2023") had been retracted from the journal due to a serious breach of journal authorship policies and of publication ethics. We regret any oversight in not recognising this retraction earlier. We have promptly removed the retracted reference and replaced it with a relevant current reference:<br><br>70. Amin, N. and Song, H. (2022) 'The role of renewable, non-renewable energy consumption, trade, economic growth, and urbanization in achieving carbon neutrality: A Comparative Study for South and East Asian countries', Environmental Science

	and Pollution Research, 30(5), pp. 12798–12812. doi:10.1007/s11356-022-22973-2.<br><br>Additionally, we have ensured that all changes to the reference list are clearly documented in the revised manuscript with the track changes.
**Comments from Editorial Office**	
Editor's Comment #1	***Response by Authors to Editor's Comment #1***
One or more of the reviewers has recommended that you cite specific previously published works. Members of the editorial team have determined that the works referenced are not directly related to the submitted manuscript. As such, please note that it is not necessary or expected to cite the works requested by the reviewer.	We appreciate the editorial team's clarification and thank you for your feedback regarding the reviewer's recommendation to cite specific previously published works in our manuscript.<br><br>We have thoroughly analysed the suggested published works to cite with the scope of our study and decided to cite one most relevant published work [Mohamed Yusoff et al. (2023)] for the current study (Refer from line number 80 to 85) by refraining from incorporating the rest of the two published works [Majekodunmi et al. (2023) and Shaari et al. (2023)] which are not directly align with the comprehensive scope of our study.
**Review Comments to the Author**	
Reviewer #1 Comment 1	***Response by Authors to Reviewer Comment 1***
I'm pleased with the revisions made by the authors; their hard work deserves credit. I'd like to contribute my thoughts to this paper.	Thank you for your kind words and encouragement regarding the revisions made to our manuscript. We sincerely appreciate your feedback and insightful comments, which have played a pivotal role in improving our manuscript to its current stage.
In Page 4, line 79 please cited the following references.<br><br>Majekodunmi, T.V., Shaari, M.S., Zainal, N.F., Harun, N.H., Ridzuan, A.R., Abidin, N.Z., Abd Rahman, N. (2023). Gas consumption as a key for low carbon state and	Thank you for your suggestion regarding the citation of references.<br><br>After careful consideration and in alignment with the editorial team's guidance, we have reviewed the suggested references. We have decided to cite the most relevant work,

its impact on economic growth in Malaysia: ARDL approach, International Journal of Energy Economics and Policy, 13(3), 469-477. http://dx.doi.org/10.32479/ijeep.14134<br><br>Shaari, M.S., Majekodunmi, T.B., Zainal, N.F., Harun, N.H., & Ridzuan, A.R. (2023). The linkage between natural gas consumption and industrial output: New evidence based on time series analysis, Energy, 284, 129395. https://doi.org/10.1016/j.energy.2023.129395<br><br>Mohamed Yusoff, N.Y., Ridzuan, A.R., Soseco, T., Whajoedi, Narmaditya, B.S. & Ann, L.C. (2023). Comprehensive outlook on macroeconomic determinants for renewable energy in Malaysia. Sustainability, 15, 3891. https://doi.org/10.3390/su15053891	Mohamed Yusoff et al. (2023), which discusses macroeconomic determinants for renewable energy, as it aligns closely with the comprehensive scope of our study (Refer from Line number 80 to 85).<br><br>The paragraph added is, “In Malaysia, several studies have implied the significant role of natural gas consumption in driving economic growth while promoting environmental sustainability. Natural gas, comparing to oil and coal, emits lower levels of CO2, positioning it as a crucial energy source for mitigating environmental degradation and supporting transitions towards cleaner energy”. Regrettably, we have refrained from incorporating the remaining references, Majekodunmi et al. (2023) and Shaari et al. (2023), as they do not directly relate to the specific variables and global analysis discussed in our manuscript.<br><br>We appreciate your understanding and constructive feedback throughout this process. Your insights have been instrumental in shaping our manuscript.
Reviewer #1 Comment 2	***Response by Authors to Reviewer Comment 2***
How does understanding causality provide novelty to your studies? Add your justification in Page 5 Line 92	Thank you for bringing this point to our attention. We recognise the importance of adding the understanding of causality to provide the novelty to our study.<br><br>We have incorporated the following paragraph in page 5 from line number 99 - 111 to address this concern.<br><br>“Understanding the causality enhances the novelty of our study by revealing the intricate relationship between economic growth, renewable and non - renewable energy consumption, and CO2 emissions trough sophisticated techniques like wavelet coherence and Granger causality analysis. This approach transcends simple correlation, offering a dynamic perspective on how these variables interact over time. By differentiating between unidirectional and

	bidirectional causality patterns and examining dynamics across global income levels, our study fills a substantial gap in the literature. It equips policy makers with actionable insights to design targeted interventions for sustainable development and climate mitigation efforts. This comprehensive understanding of causality dynamics significantly enriches the existing body of knowledge and enhances the effectiveness of policy making in addressing pressing environmental challenges."<br><br>We believe this clarification explain the novel contributions of our research effectively.
Reviewer #1 Comment 3	***Response by Authors to Reviewer Comment 3***
Please add the summary of literature gap at the end of section 2: literature review (Page 9 Line 192)	Thank you for your suggestion regarding the addition of a summary of the literature gap.<br><br>We appreciate the importance of clearly delineating the gaps in existing literature.<br><br>As suggested, we have added a summary of the literature gap at the end of section 2 (Refer the line number 216 to 230 in page 9 and page 10).<br><br>The included paragraph as follows,<br><br>"In culmination of the literature review, critical lacunae emerge in the existing body of research, notably pertaining to the dearth of studies that systematically categorise economic pathways across income levels using wavelet methodologies. This gap underscores the necessity for comprehensive analyses that not only employ advanced analytical techniques but also encompass diverse country contexts to elucidate the exact dynamics between economic growth, energy consumption and CO_2 emissions. Furthermore, despite the considerable scholarly attention devoted to the Environmental Kuznets Curve hypothesis and the relationship between economic growth and CO_2 emissions, there persists a notable absence of comprehensive

	assessments that encompass temporal dynamics and diverse country contexts. Additionally, the literature review reveals a conspicuous gap in the examination of the role of renewable energy utilization in mitigating CO2 emissions, particularly in regions abundant in renewable resources. Addressing these gaps is imperative to inform evidence-based policy interventions aimed at fostering sustainable development and mitigating adverse effects of climate change."
Reviewer #1 Comment 4	***Response by Authors to Reviewer Comment 4***
I suggest including the explanation of your model (the foundation and the expansion) into your version. Explain the expected sign as well between iv and dv. This should be placed in the earlier part of the Methodology section, Page 10.	Thank you for your insightful suggestion. When analysing the causality, we have analysed the expected sign for the variables based on the previous studies.<br><br>We have revised the manuscript to incorporate an explanation of our model, including the expected signs between independent variables (IV) and dependent variables (DV), with a supporting table (Table 2) to give more understating.<br><br>Kindly refer to the line number 254 to 260 in page 11 & 12.
Reviewer comment 05	***Response by Authors to Reviewer Comment 05***
Please enhance further your policy recommendations especially what are the best policies can be suggested from the causality test.	Thank you for your valuable suggestion.<br><br>In response, we have revised the manuscript by expanding the policy recommendations section to include actionable measures derived from the causality analyses. These include promoting renewable energy adoption, investing in sustainable technologies, strengthening regulatory frameworks, implementing urban transportation policies to reduce emissions and arranging international collaborations to address global environmental challenges.<br><br>Kindly refer to the line number 775 to 801 in page 44 & 45.

	We hope these enhancements to the policy recommendations section based on the causality test of the current study will provide valuable insights to guide policymakers and researchers toward sustainable decision making.

It would be a valuable opportunity for our manuscript to be considered for publication in PLOS ONE. We eagerly anticipate your feedback on our submission and remain available to address any additional inquiries or comments you may have at your convenience.

Thank you for your time and consideration.

Sincerely,
Prof. Ruwan Jayathilaka
Corresponding Author.
Professor / Head - Department of Information Management,
SLIIT Business School, Sri Lanka Institute of Information Technology,
New Kandy Road, Malabe, Sri Lanka.
E-mail: ruwan.j@sliit.lk

Understanding the Interplay of GDP, Renewable, and Non-Renewable Energy on Carbon Emissions: Global Wavelet Coherence and Granger Causality Analysis

Yuganthi Caldera[1], Tharulee Ranthilake[1], Heshan Gunawardhana[1], Dilshani Senevirathne [1], Ruwan Jayathilaka[2,*], Nilmini Rathnayake [3], Suren Peter [4]

[1] SLIIT Business School, Sri Lanka Institute of Information Technology, New Kandy Road, Malabe, Sri Lanka.

[2] Head - Department of Information Management, SLIIT Business School, Sri Lanka Institute of Information Technology, New Kandy Road, Malabe, Sri Lanka.

[3] Lecturer, SLIIT Business School, Sri Lanka Institute of Information Technology, New Kandy Road, Malabe, Sri Lanka.

[4] Dean, SLIIT Business School, Sri Lanka Institute of Information Technology, New Kandy Road, Malabe, Sri Lanka.

[*] Corresponding author
E-mail: ruwan.j@sliit.lk

Understanding the Interplay of GDP, Renewable, and Non-Renewable Energy on Carbon Emissions: Global Wavelet Coherence and Granger Causality Analysis

Abstract

This study examines the causality of Per Capita Gross Domestic Production (PGDP), Renewable Energy Consumption (REC), and Non-Renewable Energy Consumption (NREC) on Carbon dioxide (CO_2) emissions at the global level utilising data gathered from 1995 to 2020 across various countries categorised based on income levels as High, Low, Upper Middle and Lower Middle and analysed through wavelet coherence. The findings reveal both bidirectional and unidirectional causality between the variables which have evolved. Globally, a bi-directional relationship is observed with a positive correlation between PGDP and NREC and in contrast, a negative correlation with REC. Furthermore, the analysis highlights varying causalities between CO_2 emissions and PGDP, except for high-income and lower-middle-income country categories, all other shows one-way causality in different periods in the short term. Moreover, CO_2 and REC, show unidirectional causality throughout the short-term, exceptionally medium & long term have both unidirectional and bidirectional causalities across all country categories with a positive correlation. In contrast, CO_2 and NREC depict similar causalities to REC, however, with a negative correlation. A cross-country analysis was performed between CO_2 and PGDP, CO_2 and REC, and CO_2 and NREC using Granger causality which shows mixed relationships. The findings hold significant implications for policymakers, providing valuable insights into the trade-offs between economic growth, energy consumption, and carbon emissions.

Keywords: CO_2 emissions, Economic Growth, Renewable Energy, Non-Renewable Energy, Wavelet Coherence, Income Levels, Causality Analysis

Introduction

Currently, the world faces a pressing challenge in the form of extreme climate fluctuations driven by global warming, with CO_2 emerging as a prominent contributor among various greenhouse gases [1-6]. CO_2, primarily emitted from activities such as fossil fuel combustion, cement manufacturing and transportation stands out as the foremost contributor to global warming. Its potent heat-trapping properties and significant concentration within the Earth's atmosphere contribute to the escalating impacts of climate change [7]. Understanding the sources and dynamics of CO_2 emission is crucial for devising effective strategies to mitigate climate change and its adverse effects.

As economies expand, the demand for energy rises, often leading to increased reliance on fossil fuels for energy production. This growth-driven rise in energy consumption is closely linked to rising CO_2 emissions, posing significant challenges to achieving sustainable development [8]. Particularly in developing nations, non-renewable energy sources are often overused or mismanaged due to various socio-economic factors. Consequently, addressing the dual imperative of economic growth and environmental sustainability has become a focal point for both researchers and policymakers worldwide. Studies exploring the Environmental Kuznets Curve hypothesis have provided valuable insights into the complex relationship between economic growth and environmental degradation. The same relationship is confirmed by studies conducted for 11 newly developed countries and 83 selected countries as concrete evidence for CO_2 emission and GDP growth [9, 10]. However, regulating CO_2 emissions presents a multifaceted challenge due to its close association with energy production. As a

result, preventive measures targeting CO_2 emissions could potentially hinder economic growth, particularly in developing nations [11]. Numerous empirical studies across diverse nations have consistently revealed a direct correlation between economic growth and CO_2 emissions by utilizing sophisticated econometric techniques for countries such as Pakistan [12], China [13], Kazakhstan [14], Indonesia [15], Turkey [16], Nigeria [17], Egypt [11], and Bangladesh [18].

Renewable energy sources, including wind, solar, geothermal, wave and tidal power are anticipated to be the fastest-growing sectors within the energy industry. This growth is driven by the recognition of renewable energy's crucial role in addressing energy security and climate change issues. In Malaysia, s~~S~~everal studies have implied the significant role of ~~, in Malaysia, the critical impact of~~ natural gas consumption ~~has driven~~in driving economic growth~~,~~ while ~~contributing to~~promoting environmental sustainability. Natural gas, c~~C~~omparing to oil and coal, ~~natural gas has a~~ emits lower levels of CO_2, positioning it as a crucial ~~emission which highlights as an essential~~ energy source for~~to~~ mitigating~~e~~ environmental degradation and supporting ~~clean energy~~ transitions towards cleaner energy [19]. Notably, China's substantial investment of over 170 billion USD in clean energy surpasses the European Union and the United States, signalling a significant shift in global energy investments [20]. This investment highlights the pivotal role of renewable energy in driving economic development and reducing greenhouse gas emissions on a global scale. Despite the promising potential of renewable energy consumption, its widespread adoption is expected to bring both opportunities and challenges. While renewable energy consumption holds promise for meeting future energy demands and reducing carbon dioxide emissions, it also poses challenges such as the need for infrastructure development and grid integration. Additionally, emerging technologies like hydrogen or electronic (battery-driven) based vehicles present promising alternatives to traditional fossil fuels powered transportation, however, their widespread adoption requires overcoming technical and economic barriers [21-23].

The main objective of this study is to examine the causality of the variables CO_2, PGDP, REC and NREC considering variations over a 26-year period in order to address a significant knowledge gap in the existing literature. Understanding the causality enhances ~~in our study introduces~~the novelty of our study by ~~unrevealing~~revealing the intricate relationship between economic growth, renewable and non - renewable energy consumption, and CO_2 emissions trough sophisticated techniques like wavelet coherence and G~~g~~ranger causality analysis. This approach ~~goes beyond~~transcends simple correlation, offering a dynamic perspective on how these variables ~~influence each other~~interact over time. By differentiating between unidirectional and bidirectional causality patterns and examining ~~considering the~~dynamics across global ~~context across~~ income levels, ~~this~~our study ~~study~~ fills a ~~significant~~substantial gap in the literature.~~,~~ ~~providing~~It equips policy makers with actionable insights to design targeted interventions for sustainable development and climate mitigation efforts. This comprehensive understanding of causality dynamics ~~contribute~~ significantly~~ubstantially to the~~enriches the existing body of knowledge and enhances the effectiveness of policy making in addressing~~, pressuring the~~pressing environmental challenges. ~~Specially~~Further, researchers intend to conduct an income category wise analysis to investigate variations between different country categories, representing a recent addition to the literature. Thus, t~~T~~his study shows a prominent value addition in three keyways.

Firstly, researchers employ a novel methodology of Wavelet coherence, which provides a unique perspective on the relationship and causality between CO_2, PGDP, REC and NREC. Unlike previous studies, Wavelet coherence allows a more nuanced analysis by simultaneously considering both time and frequency domains, providing insights into the dynamic interactions between these variables over time. This methodology helps to examine the linkage between the variables across short-term, medium-term and long-term time scales with the direction of arrows for the period of 1995 to 2020. Further, the methodology shows correlation variations

emphasising whether it is high, medium or low between variables with the frequency scale as well. Additionally, Granger causality was adopted to analyse the causality between the variables for each individual country to enhance the findings shown through wavelet coherence.

Secondly, this study adopts a global approach covering 161 countries categorised based on the income level. While existing literature has predominantly focused on individual countries, organisations, and different regions, this comprehensive global study based on income level provides a unique insight into the relationship between economic growth, energy consumption, and CO_2 emissions.

Thirdly, the study presents a summarisation of graphs plotted for the world and all income categories through wavelet coherence into one output. This output showcases the directions and relationships between variables across different time scales; short term, medium term and long term, highlighting correlation variations over the 26-year period by allocating a five-year time range. Hence, this study offers valuable insights for policymakers, researchers, and stakeholders in understanding and addressing the complex dynamics of CO_2 emissions and energy consumption.

Further, it sheds light on the role of technological advancements in reducing CO_2 emissions and the impact of increased utilisation of renewable energy sources on mitigating CO_2 emissions. This research serves as a resource for inventors, policymakers, and entrepreneurs, providing them with predictive insights into how innovations in technology and policy can contribute to the reduction of CO_2 emissions and the preservation of the environment. Through a comprehensive understanding of the factors driving CO_2 emissions and the potential solutions available, stakeholders can make informed decisions and take proactive steps toward promoting sustainability and mitigating climate change.

The subsequent sections of the article are structured as follows: a review of existing literature, followed by an explanation of the data and methodology, then the presentation of findings and discussion, and finally, the overall conclusion of the study.

Literature review

Fossil fuel combustion is a major contributor to the global issue of climate change, releasing a large amount of CO_2 emissions [24]. A similar study done using panel Granger causality analysis across various income categories such as high-income, upper-middle-income, lower-middle-income, and low-income has identified bidirectional causality between CO_2 emission and energy consumption. It further reveals a unidirectional causality between GDP and CO_2 emission in upper-middle-income economies, A unidirectional causality is identified in lower-middle-income economies and both the long and short run, a bidirectional causality is displayed in low-income economies. [25, 26]. Furthermore, the connection between non-renewable energy and CO_2 emission is shown in the results that changes in CO_2 emission will lead to changes in non-renewable energy consumption with a negative relationship [27]. Additionally, analyses conducted on G-20 countries between 2010 and 2019 have highlighted the positive and significant impact of GDP and non-renewable energy consumption on CO_2 emissions, whereas a negative and significant effect was observed from renewable energy sources [28]. Notably, despite the growing body of literature in this area, only a few studies have examined income level based categorisation of economies using wavelet methodology.

A study conducted in Saudi Arabia investigated the consumption of non–renewable energy and its associated factors, which have contributed to an increase in CO_2 emissions [29]. Moreover, the study examines the relationship between the income level of a country and its CO_2 emission, and it confirms the Environmental Kuznets Curve hypothesis. This hypothesis

suggests that increased income can initially lead to higher carbon emissions, and it is possible to mitigate these emissions without compromising economic growth [30]. Additionally, trade openness helps to reduce pollution levels and highlights the need for advanced technologies and renewable energy sources in environmental management. A study utilising wavelet coherence to investigate the relationship between economic growth and environmental pollution found that G7 countries exhibit higher coherence in the short term, with increased economic growth correlating with higher pollution levels [31]. The study suggests that G7 countries should strengthen economic cooperation and design efficient policy instruments, such as implementing short-term taxation on those who emit carbon dioxide on a mass scale in order to address the environmental challenges.

In identifying the asymmetric causality in greenhouse gas emissions in Saudi Arabia, it was found that, unidirectional asymmetric causality results from both positive and negative changes in CO_2 emissions to REC [32]. In both the short-term and long-term periods, the same outcome of asymmetric causality resulting from the positive and negative shocks of the real GDP to REC occurred. Similarly, analyses conducted in Algeria, a country which is rich in renewable energy resources, highlight the low share of renewable energy utilization despite its abundance. Studies in this context demonstrate a significant correlation between economic growth and CO_2 emission [33]. Furthermore, wavelet coherence results indicate that economic growth is the leading variable influencing both CO_2 emission and energy consumption suggesting a co-movement between economic growth and CO_2 emissions. Interestingly, economic growth appears to have a negative impact on energy consumption but positively affects CO_2 emissions, as observed in Sub-Saharan Africa [34]. These studies suggest that CO_2 emissions may stimulate economic growth and they do not necessarily drive energy consumption, highlighting the complex dynamics between economic development, energy use, and environmental outcomes.

Countries like Oman, Qatar, and Saudi Arabia show an inverted U-shaped Environmental Kuznets Curve, where CO_2 emissions initially increase with GDP growth before eventually declining [35]. To address this trend, it is recommended to implement necessary economic and social policies that are aimed at reducing CO_2 emissions while simultaneously fostering economic growth. Similarly, a study conducted in Thailand using Wavelet and Granger analysis techniques showed that economic growth leads to CO_2 emission, and energy consumption and CO_2 emissions mutually predict each other [36]. The same findings have strengthened the awareness of energy efficiency among citizens to minimise the negative impacts of CO_2 emissions on the environment. In Ghana, the energy sector heavily relies on fossil fuels which have a detrimental effect on CO_2 emissions [37]. The results of the causality studies support a unidirectional relationship between energy consumption and economic growth. The level of penetration of renewable energy has not reached the point to mitigate CO_2 emissions. Consequently, Ghanaian policymakers should implement policies focused on sustainable development, such as increasing the usage of renewable energy.

The reliance on coal, oil, and natural gas has emerged as the primary driver of non-renewable energy consumption, posing sustainability challenges due to the finite nature of these resources [38]. Continuous reliance on non-renewable energy sources risks resource depletion in the long term. Moreover, empirical findings suggest a bidirectional causality between renewable and non-renewable energy sources and economic growth in the short and long run [39]. Therefore, managing the use of non-renewable energy sources may not be sufficient to foster sustainable economic growth.

In culmination of the literature review, critical lacunae emerge~~s~~ in the existing body of research, notably~~e~~ per–t~~r~~aining to the dearth of studies that systematically categorises~~es~~ ~~,~~ economic pathways ~~on~~across income levels using wavelet methodologies. This gap underscores the necessity for comprehensive analyse~~i~~s that not only employ advanced

analytical techniques but also encompass diverse country contexts to elucidate the ~~nuanced~~exact dynamics between economic growth~~-~~, energy consumption and CO_2 emissions.~~,~~ Further-more, despite the considerable scholarly attention~~dance~~ devoted to the E~~e~~nvironmental Kuznets C~~c~~urve hypothesis and the relationship between e~~E~~conomic growth and CO_2 emissions, the~~i~~re persists~~ence~~ ~~,—~~a notable absence of comprehensive assessments that encompass temporal~~ry~~ dynamics and diverse country contexts. Additionally, the literature review reveals a conspicuous gap in the examination of the role of renewable energy utilization in mitigating CO_2 emissions, particularly in regions ~~aban~~abundant~~doned~~ in renewable resources. Addressing these gaps is imperative to inform ~~evidence based~~evidence-based policy interventions aimed at fostering sustainable development and mitigating adverse effects of climate change.

Data and Methodology

Data

The study encompasses annual data collected from 161 countries spanning the years 1995 to 2020. Data have been extracted through secondary data presented in S1 Appendix, outlining the variables of interest. The relevant source and measure of the variables taken are illustrated in Table 1.

Table 1: Data sources and definition of variables

Variables	Measure	Source
CO_2 Emissions	Metric tons Per Capita of CO_2	https://data.worldbank.org/indicator/EN.ATM.CO2E.PC
PGDP	Per capita GDP (Current US$)	https://data.worldbank.org/indicator/NY.GDP.PCAP.CD
REC	% of total final energy consumption	https://data.worldbank.org/indicator/EG.FEC.RNEW.ZS
NREC	100 - % of total final energy consumption	https://data.worldbank.org/indicator/EG.FEC.RNEW.ZS

Source: Authors' Compilation.

The selected 161 countries comprise of 45 high-income countries, 20 low-income Countries, 51 low-middle-income countries, and 45 upper-middle-income countries to have a comprehensive analysis to investigate the causality among the variables CO_2, PGDP, REC and NREC.

Methodology

The study employs Wavelet coherence methodology to assess causality and correlation within the time series dataset utilising R-Software. The inception of this methodology is explained by P. Goupillaud [40], and currently, the wavelet approach has been discussed in many studies [33, 36]. Fourier analysis is the root of the Wavelet coherence method, which is used to obtain results through graphical representations, but it is not an efficient way to detect sudden fluctuations. Nevertheless, the Wavelet approach has overcome these limitations with the existence of a zero mean based on a limited period [41, 42].

This section serves to introduce the foundational aspects and expansion of the current study model. The foundation of current study model is grounded in previous research findings,

guiding our expectations regarding the signs of relationships between independent variables and dependent variables. Specifically,

Table 2 comprises the past ~~literatures~~studies emphasising the expected sign of the variables.

Table 2: Variables ~~and supporting~~and supporting past ~~studies~~studies.

**Variable**	**Past Studies**
CO_2 ⟷ PGDP (+)	Attiaoui, Toumi [43]; Peng, Tan [44]; Mohamed Yusoff, Ridzuan [19]; Dharmapriya, Edirisinghe [45] Raihan, Voumik [46]; Banday and Aneja [47]
CO_2 ⟷ REC (-)	Le [27]; Attanayake, Wickramage [48]; Ponce and Khan [49]
CO_2 ⟷ NREC (+)	Phatchapa Boontome [50]; Dogan and Seker [51]; Dogan and Seker [52]

Source: Authors' Compilation.

A Wavelet, generated by the function $\psi^{a,b}(x)$, with contractions, a and translation, b can be statistically denoted as follows (ψ denotes Morlet Wavelet function),

$$\psi^{a,b}(x) = |a|^{-\frac{1}{2}} \psi\left(\frac{x-b}{a}\right) \quad (1)$$

Previous studies have emphasised the advantages of using wavelet coherence methodology. Data filling is not required unlike other linear and nonlinear methodologies, and results can be obtained through graphical representation without relying on numerical statistics. Moreover, the fluctuations of the periods can also be categorised based on long term, medium-term, and short-term and based on the frequencies as well [53]. Considering finance and economics, medicine, tourism and financial development, the Wavelet coherence approach has made a prominent step [54, 55]. The Wavelet coherence method is a bivariate analysis which explains why the other variables lead the second with different time ranges. This is more advanced than approaches such as Granger causality since it can explain the direction and the strength of the causal relationship between the variables. Two-time series can be evaluated in wavelet to realise what variable impacts the other.

As mentioned above in previous empirical studies, wavelet analysis is significant in time-frequency analysis which means it provides the frequency in time representations for the causality of variables. Moreover, this provides multiscale analysis which can capture the causality of variables with different frequencies.

The Granger causality test was adopted for each individual country to enhance the results gained through Wavelet coherence analysis [56]. This study uses VAR Granger analysis to evaluate the causality between CO_2 and PGDP, CO_2 and REC, and CO_2 and NREC for each country utilizing Stata software. This approach has been done in many studies [57]. First, the study ran the Augmented Dickey-Fuller test to check the stationarity of the variables of the two-time series. The two stationary covariance variables are X and Y. This shows the causality for each individual, explaining that the Xi′t variable causes Yi, t if it's better able to predict Yi,t by employing all the required information of variables, which is compared to the use of information except Xi,t for each individual. The assumption would be the model is linear, therefore using the time-stationary VAR representation to each cross-sectional unit i and time period t.

$$Y_{i,t} = \sum_{k=1}^{p} \beta_i \, Y_{i,t-k} + \sum_{k=0}^{p} \Theta_k \, X_{i,t-k} + u_{i,t} \qquad (2)$$

There u is normally distributed with $u_{i,t} = \alpha_i + \varepsilon_{i,t}$, where the number of lags is denoted as p. Under the assumption the autoregressive coefficients β_k and the regression coefficients θ_k's are constant for $k \in [1, N]$. The equation depicts that u is normally distributed, where Y is the dependent variable (i and t denote the country and time, respectively), X is the independent variable, ui, t denotes the error term, and k is the number of lags.

Results and Discussion

The descriptive statistics of the current study are presented in Table 3~~2~~. The dataset comprises 4186 observations, with 1170, 520, 1170, and 1326 observations corresponding to high-income, low-income, upper-middle-income, and lower-middle-income groups respectively. The average CO_2, emissions at the global level for, high income, low-income, upper-middle-income, and lower-middle-income groups are 3.97 metric tons per capita, 9.181 metric tons per capita which is the highest, 0.266 metric tons per capita the lowest, 3.445 metric tons per capita, and 1.279 metric tons per capita respectively. The average values of PGDP are US$ 11,359, US$ 33,189 the highest, US$ 710 the lowest, US$ 5,177, and US$ 1,728 at the global level for, high-income, low-income, upper-middle-income, and lower-middle-income respectively. Considering REC and NREC, it would calculate the 100% of final energy consumption, and the average values of REC are 34.87% for the world, 16.5% for high income, 75.6% for low income, 21.48% for upper-middle income, and 46.94% for lower-middle income. This suggests that low middle-income countries have the highest proportion of renewable energy consumption, while high-income countries have the lowest.

Table 3~~2~~: Summary of descriptive statistics of variables

**Country Category**		**Variables**			
		**CO_2**	**PGDP**	**REC**	**NREC**
**Global**	Obs	4186	4186	4186	4186
	Mean	3.967	11358.670	34.877	65.123
	SD	5.175	19489.530	30.428	30.428
	Min	0.022	99.757	0.002	1.660
	Max	47.657	179467.500	98.340	99.998
**High Income**	Obs	1170	1170	1170	1170
	Mean	9.181	33188.830	16.503	83.497
	SD.	6.636	26009.210	17.060	17.060
	Min	1.355	826.973	0.010	17.210
	Max	47.657	179467.500	82.790	99.990
**Lower Income**	Obs	520	520	520	520
	Mean	0.266	709.851	75.601	24.399

Country Category		Variables			
		CO_2	PGDP	REC	NREC
	SD	0.525	1169.685	26.689	26.689
	Min	0.022	99.757	0.580	1.660
	Max	3.099	11304.640	98.340	99.420
**Upper Middle Income**	Obs	1170	1170	1170	1170
	Mean	3.445	5176.533	21.475	78.525
	SD.	2.730	3137.035	19.281	19.281
	Min	0.470	252.975	0.002	9.880
	Max	15.341	19849.720	90.120	99.998
**Lower Middle Income**	Obs	1326	1326	1326	1326
	Mean	1.279	1727.602	46.945	53.055
	SD	1.485	1337.658	28.413	28.413
	Min	0.050	107.393	0.060	5.230
	Max	7.751	9225.845	94.770	99.940

Note: Obs, SD, MIN, and MAX define Observation, Standard Deviation, Minimum, and Maximum, respectively
Source: Authors' Compilation.

The violin plots in Fig 1 depict the dispersion of the data for CO_2 emission, PGDP, RE, and NRE for all four country categories, namely high income, low income, lower middle-income, and upper middle-income. The data are clustered together for the CO_2 emission of all the country categories except for lower-middle-income countries, indicating less variation among the data is less. The data points are distributed around the mean for low-income countries.

Considering the data dispersion of data for RE, similar to CO_2 the data has been clustered together for high income countries and upper middle-income countries. The distribution of data points around the mean is observed for low-income countries, while data is distributed below the mean for lower-middle-income countries. In RE, the median of the low-income county category is higher than other country categories, and vice versa for NRE. Further, in lower-middle-income countries, there is a uniform distribution, while in high-income and upper-middle-income countries, the distribution is below the mean. The NRE shows the exact opposite results to RE due to the perfect correlation between the two variables.

Fig 1. Violin plots of CO2 emission, GDP, RE and NRE by country category.

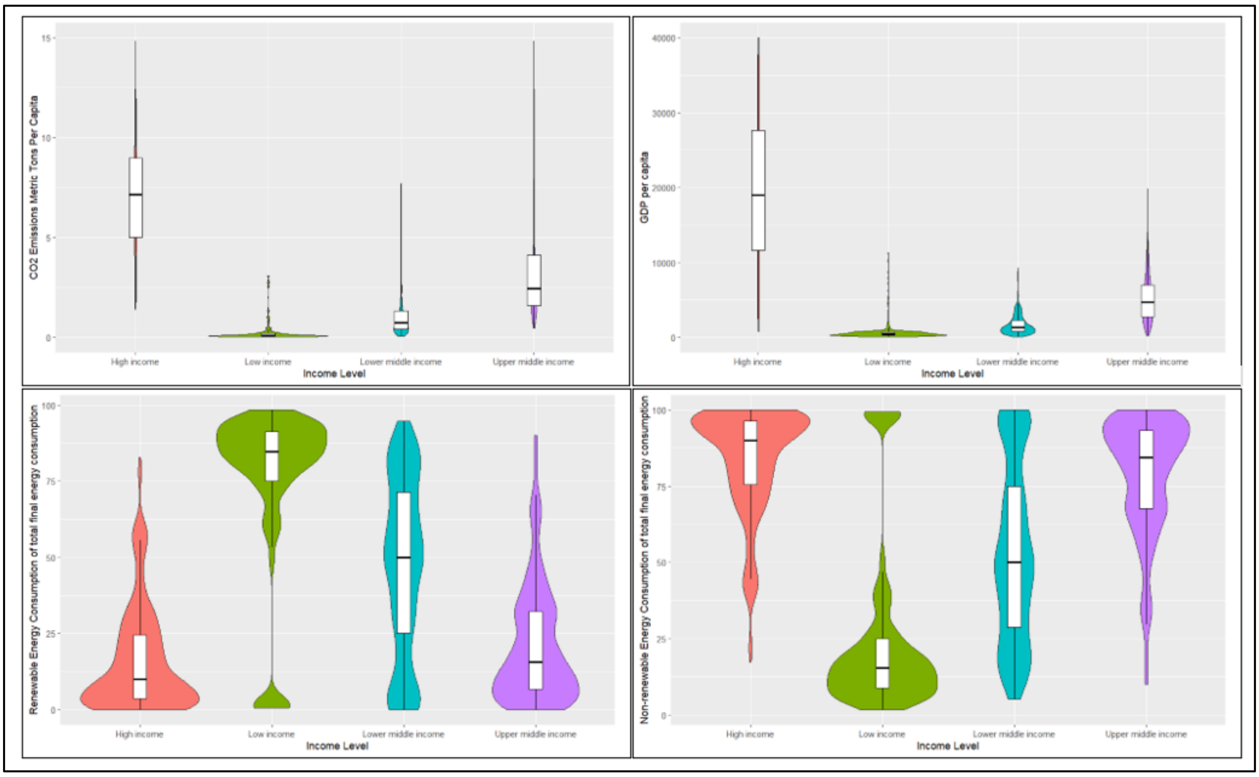

Source: Authors' Compilation.

The wavelet coherence graphs for the world and four income categories have been discussed with interpretations in this section. Table 4~~3~~ depicts the attributes related to the graph and how they have been interpreted. In this study, the first variable is considered CO_2, and the second variable is PGDP, REC, and NREC. The graphs have been plotted as CO_2 and PGDP, CO_2 and REC, and CO_2 and NREC for the global level and income category-wise.

Table 4~~3~~: Interpretation of Wavelet Coherence

Attributes	Interpretation
**Horizontal Axis**	Time period
**Vertical Axis**	Scale
**White Cone**	Cone of Influence
**Thick Black border**	95% confidence level
**Rightward arrow**	Positive relationship (In-Phase)
**Leftward arrow**	Negative relationship (Out-phase)
**Rightward up arrow**	Second variable (PGDP/RE/NREC) causes the first variable (CO_2)
**Rightward down arrow**	First Variable (CO_2) causes the second Variable (PGDP/RE/NREC)
**Leftward up arrow**	Second variable (PGDP/RE/NREC) causes the first variable (CO_2)
**Leftward down arrow**	First Variable (CO_2) causes the second Variable (PGDP/RE/NREC)
**Cold region (Blue)**	No correlation
**Warm region (Red)**	Correlation exists

Source: Authors' Compilation.

The white cone of the illustrations depicts the Cone of Influence which means the region in the time-frequency where the edge effects are significant. The 5% significance level is interpreted using the thick black border calculated using the Monte Carlo simulations. The red region signifies that there is a correlation, and the blue region explains that the correlation doesn't exist. Moreover, considering the directions rightward arrow depicts a positive correlation and in contrast left arrow depicts a negative correlation. If the rightward or leftward arrows move up, it means the second variable causes the first variable which signifies a one-way causality. Further, if the rightward or leftward arrows move downwards, it depicts that the first variable causes the second variable which also signifies a one-way causality. The arrows' movement up and down considering the path of right and left, signifies a bidirectional causality between the respective variables.

As shown in Fig 2, there is a positive correlation between CO_2 and PGDP in the short term (Scale ranging from 0-16), and there is a bi-directional relationship in 1995, 1999, 2002, 2008, 2013, 2015 and 2018 at high frequency with a significance of 5%.

Fig 2. Wavelet coherence: CO_2 vs PGDP for world

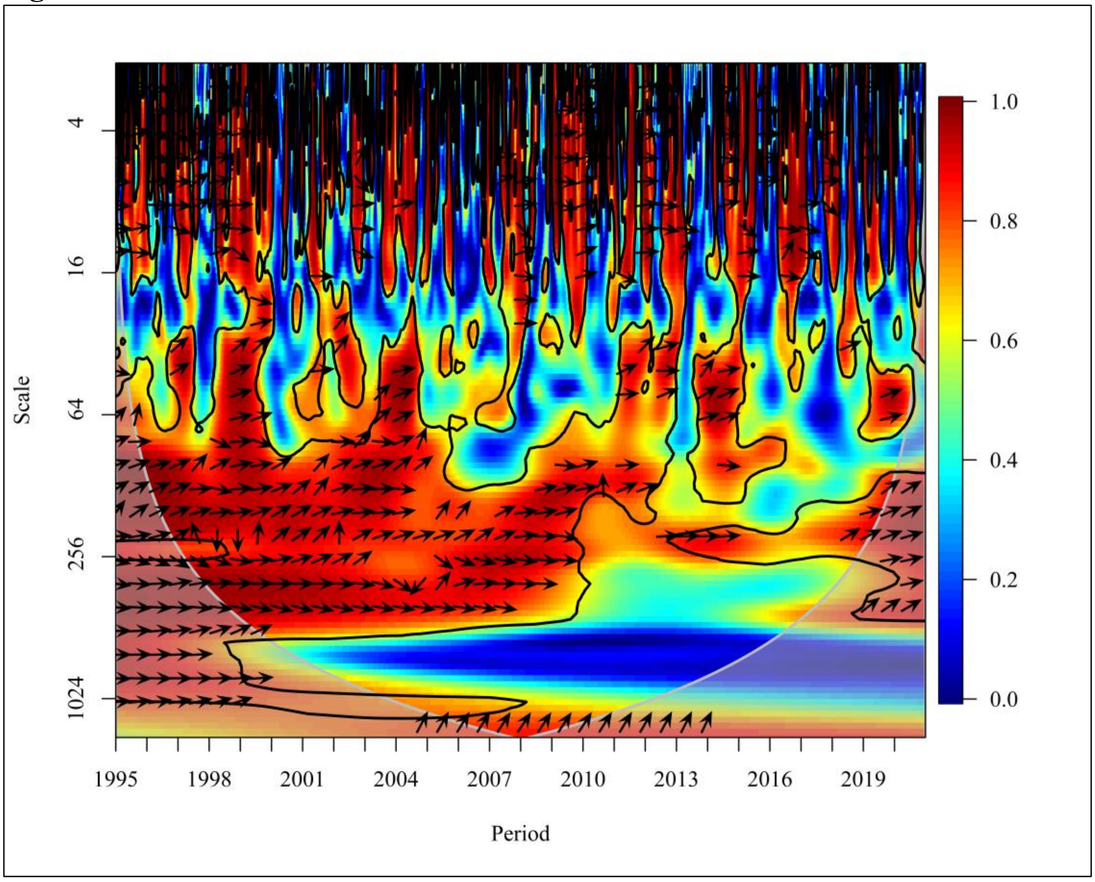

In the years 1997 and 2009, CO_2 emissions have a unidirectional causality to PGDP. In contrast, the years 1998, 2004, 2007, 2010, 2012, and 2014 show that PGDP had caused positive to CO_2 emission at 5% significance with high frequency. The period ranging from 1996 to 2019 shows a positive correlation between CO_2 and PGDP in the medium term (Scale ranging from 16-256). Further from 1995 to 1998, 2001, 2003 to 2007, and 2011 to 2012 there has been a bidirectional causality between CO_2 and PGDP with high and medium frequency at 5% significance. Thus, from 1999 to 2000, 2002,2008 to 2010, 2013 to 2014 and in the last 3 years, which is 2018 to 2020 there has been a unidirectional causality where PGDP has caused positive CO_2 emissions. In the long term (Scale from 256 – 1024), it is visible that, from 1998

to 1999 there has been a unidirectional causality in which PGDP has caused CO_2 emission and from 2000 to 2010 there has been a bidirectional causality between the variables at high frequency at 5% significance. The study done using Granger found that there is a unidirectional relationship between PGDP and CO_2 emission. When analysing in the global context there is no causality in underdeveloped countries. Additionally, a bidirectional relationship is found between CO2 emission and PGDP in economies in transition countries for the period of 1990 to 2019 [58, 59]. Representing ASEAN countries, Malaysia, Philippines, Singapore and Thailand consist of high income , lower middle income and upper middle income countries representing that there is a unidirectional relationship between CO2 and PGDP [60].

High-income level countries in the short term (Scale ranging from 0-16), have a mixed relationship between PGDP and CO_2 with high and medium frequencies, signifying that there's a positive relationship in the years 2007, 2010, 2013, 2016, and 2020 and in contrast, a negative relationship in 1999 and 2005 as shown in Fig 3. There is a cross-correlation or a bi-directional relationship between these two variables in 1999 to 2000, 2005, the second half of 2006 to 2007, 2013, and 2020 for high frequency. In the 1st half of 2006 and 2011, PGDP caused CO_2 with a negative impact with high frequency. In 2010 and 2016 PGDP positively caused CO_2 with high frequency. Further CO_2 has caused positive to PGDP in the second half of 2011. In the medium term (Scale from 2016 – 64), PGDP negatively led to CO_2 from 2000 to 2001 and 2007 to 2008 at medium frequency. Further in 2012 and 2013, PGDP led to CO_2 emissions positively. In the long term (Scale 64 - 256) effects are only shown in 1998 to 1999 where PGDP has positively caused CO_2 emission at a medium frequency. A study conducted in the USA shows that the causal flow is stronger around the period of 1910 to 2014. The study has found a bidirectional relationship between CO_2 and PGDP.

Fig 3. Wavelet coherence: CO_2 vs PGDP for High-income countries

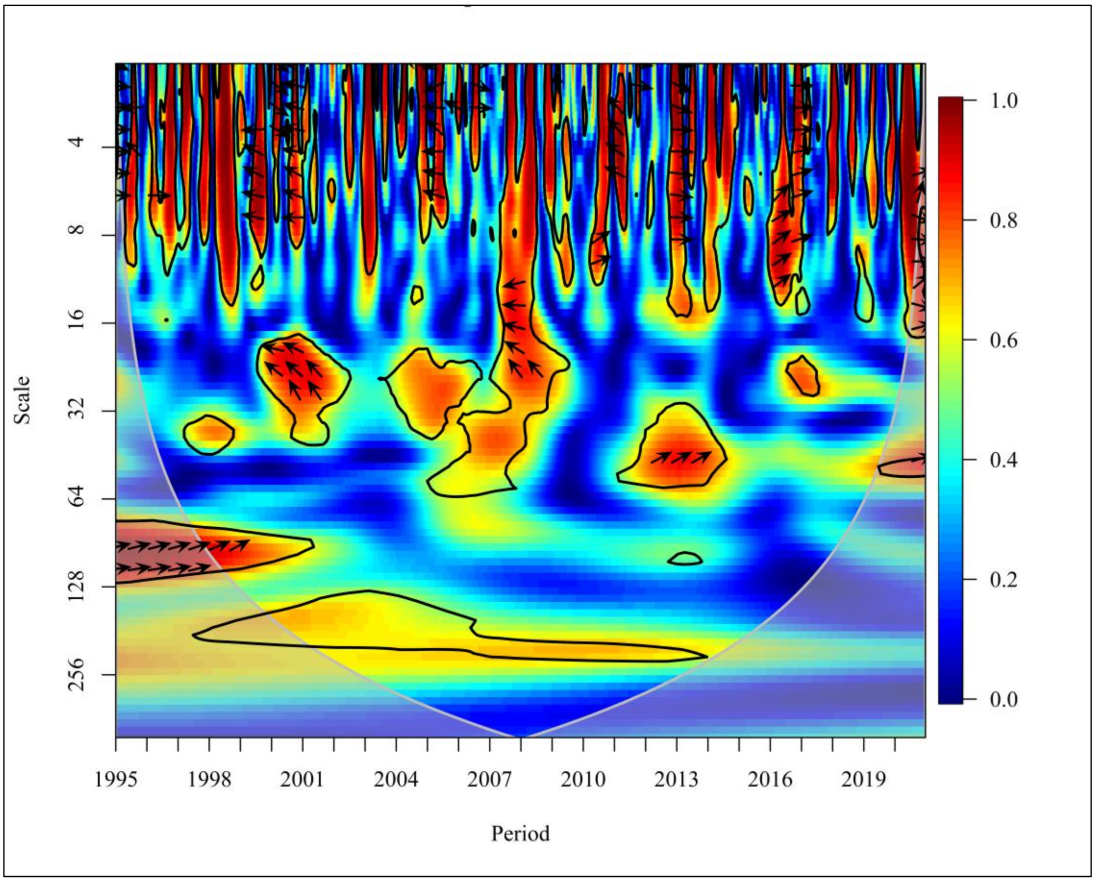

Nonetheless, the causality has a strong, positive long-run relationship between these two variables under the suggestion that the deterioration of the environment is due to economic expansion. In contrast, in the early 1990s, there was a reverse causality. Some factors for the rise in CO_2 that has been attributed can be industrialisation, energy price increments, and new technologies, while this can be disrupted by volatilities like the great depression and World War [61].

In Fig 4, Lower income classified countries have an overall positive relationship between CO_2 and PGDP in high, and medium frequencies in all three periods: short-term, medium-term, and long-term. In the short run (Scale 0-8), bidirectional causalities can be seen in 1995 to 1996 and 2008. In 2007, the first half of 2013, 2015, and 2018 PGDP caused positive CO_2 emissions at high and medium frequencies. Also, CO_2 emission has positively led to PGDP in 2004 to 2005, 2009, 2nd half of 2011, and 2019 at high and medium frequencies. In the medium term (Scale 8-32) there is a bidirectional causality between CO_2 emission and PGDP

in the years 1996 to 1997, 2005, 2007, and 2015. Years 2004, 2006, and 2008 to 2010 there is a positive causality where CO_2 has led to PGDP. Only in 2017, there is a negative relationship with a unidirectional causality from PGDP to CO_2 is visible. From 1998 to 2018 in the long-term (32 – 128) has a positive relationship between CO_2 and PGDP in high frequency.

Fig 4. Wavelet coherence: CO_2 vs PGDP for Low-income countries

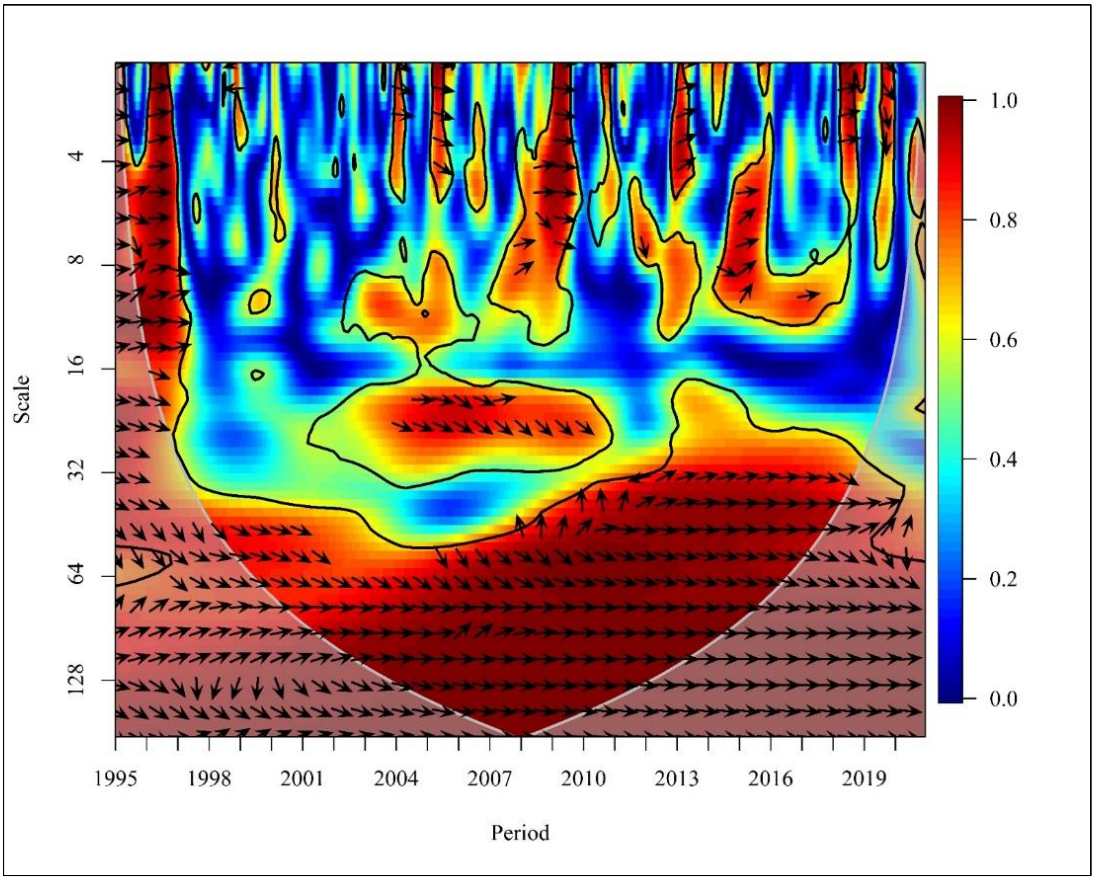

From 1998 to 2005, there has been a unidirectional causality where CO_2 has led to PGDP while from 2006 to 2017 there is a unidirectional relationship in which PGDP has led to CO_2. Again in 2018, CO_2 has led to PGDP. A study related to top oil-generating countries has categorised four countries named Congo, Guinea-Bissau, Niger, and Sudan as low-income countries which is in line with the current study. CO_2 and PGDP have shown a significant causal relationship in the short run with a positive one-way causality with CO_2 leading PGDP

for the period 2000 to 2019. In the medium term, variables have a cyclic relationship and lastly in contrast to the current study in the long run from 2013 to 2019, there is a negative correlation between PGDP leading CO_2 [62].

For the lower-middle-income countries, Fig 5 represents a mixed relationship consisting of both positive and negative for different periods. In the short run (Scale 0-16), there is a bi-directional relationship from 1996,1998,2002,2006 to 2009, 2011, 2014 to 2017 and 2020 with high frequency. In 1999, 2004, 2012 to 2013 PGDP caused CO_2 emission negatively at a high frequency.

Fig 5. Wavelet coherence: CO_2 vs PGDP for Lower Middle-Income countries

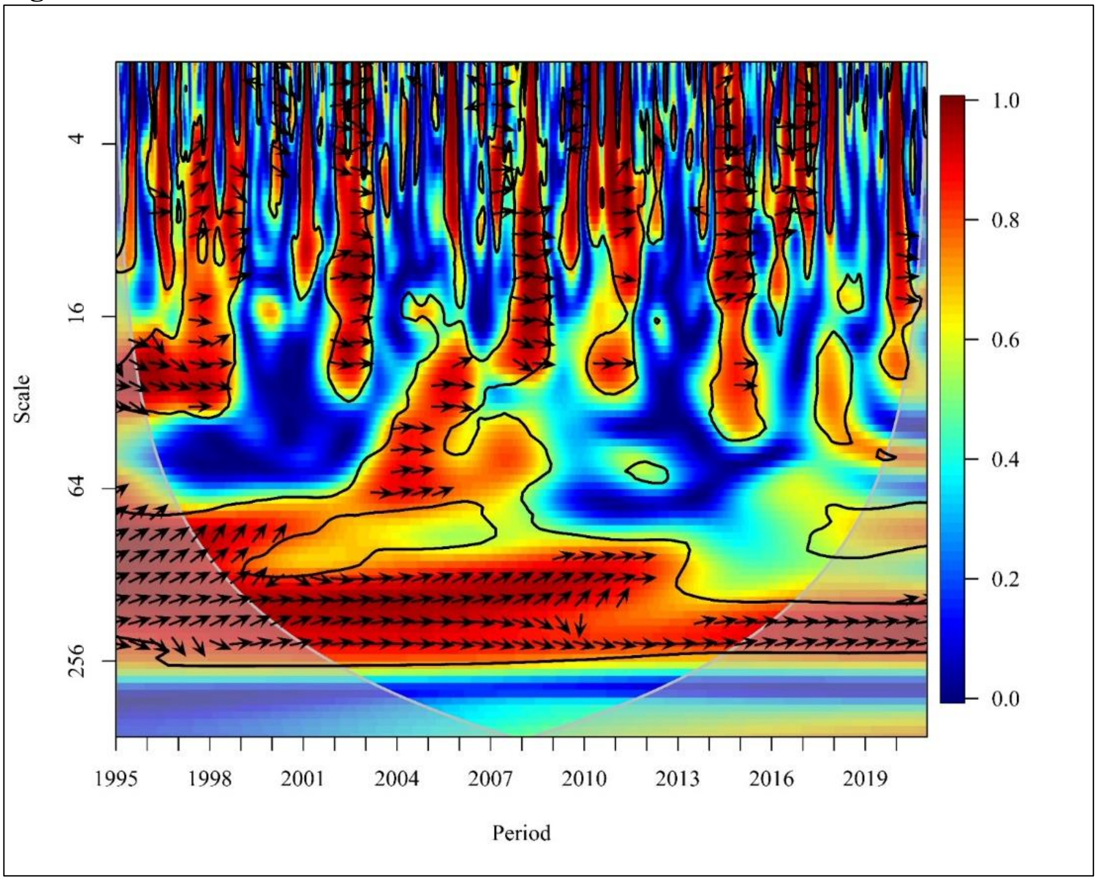

In 1997, PGDP caused CO_2 emission but in 2000, CO_2 caused PGDP positively with high frequency. In the medium term (Scale 16 – 64), a bidirectional relationship is depicted

positively in 1996 to 1998 and 2nd half of 2004 to 2006 at high frequency. A unidirectional causality is depicted in 2002, 2007 2008, 2010, and 2015 which CO_2 has positive causality to PGDP. Only in 1st half of 2004, a Positive relationship with the causality of PGDP leading to CO_2 is depicted. In the long term (Scale 64-256) from 2000 to 2012, there is a clear positive bidirectional causality. In the 1998 to 1999 and 2013 to 2016 periods, unidirectional causality is depicted positively at high frequency. A study done in Algeria which is a lower-middle-income country has shown a short-term relationship between PDGP and CO_2, from 1994 to 2011. The period from 1994 to 2006, has conveyed a positive correlation with PGDP leading, and from 2006 to 2011 CO_2 led PGDP. The study has interpreted that there is a significant movement between PGDP and CO_2 in all the time scales with PGDP leading to CO_2. Since Algeria is an oil-rich nation and mainly relies on fossil fuels, energy consumption has driven up and has impacted CO_2 because when the economy grows, the carbon emissions have been expanded [33].

In upper middle-income countries in the short run (Scale 0 – 16), there is a bi-directional relationship in 1996, 2nd half of 2000, 2nd half of 2000, 2012, and 2018 shown in Fig 6.

Fig 6. Wavelet coherence: CO_2 vs PGDP for Upper Middle-Income countries

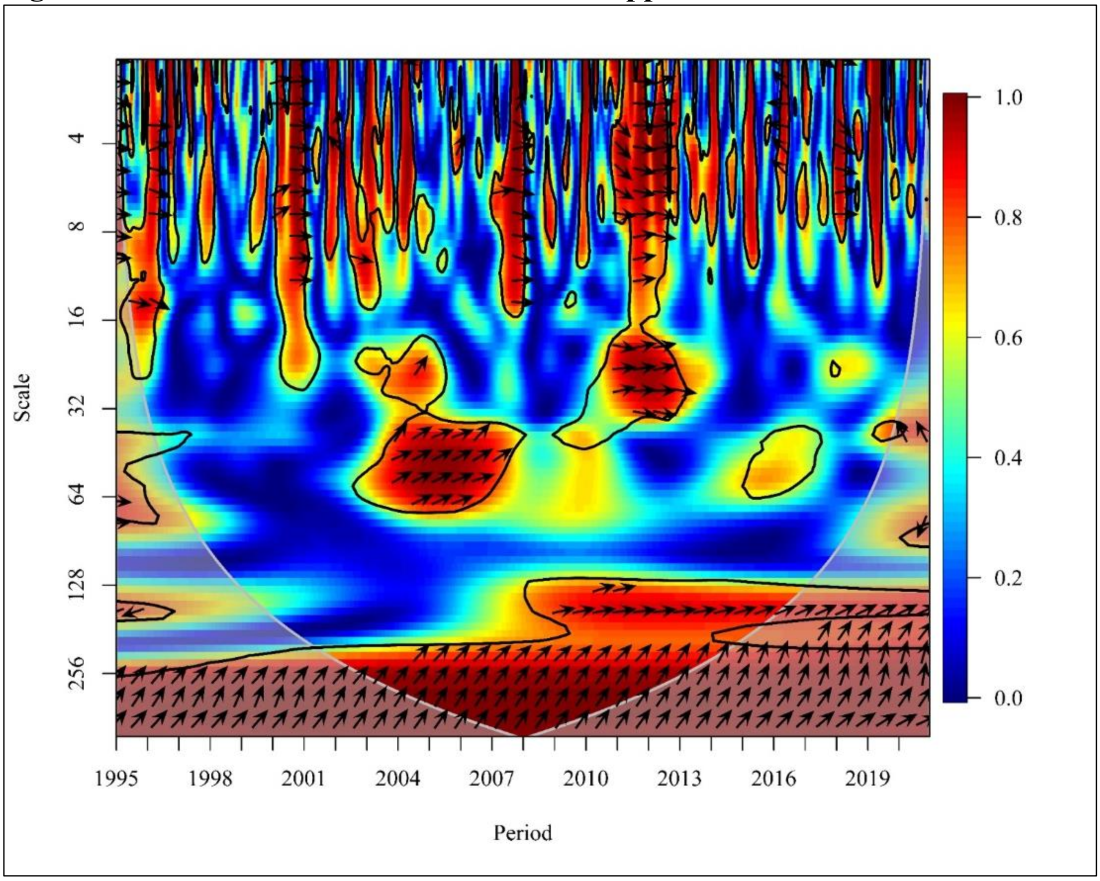

A positive relation with the causality of PGDP leading to CO_2 emission has been shown in 2000 and 2005. In 2nd half of 2002 and 1st half of 2011 depict a unidirectional relationship where CO_2 has led to an increase in CO_2. Further in 1st half of 2002 and 2016, there has been a negative relationship where PGDP has led to CO_2. In the medium term (Scale 16 – 64), a high frequency is depicted where PGDP has led to CO_2 from 2004 to 2007. In the years 2011 to 2012, there was a bidirectional relationship and in 2013 there was a unidirectional relationship where CO_2 has led to PGDP. Long-term (Scale 64 – 256) depicts a clear visual of PGDP causing CO_2 emission at a stretch from 2003 to 2016 at high frequency with a positive relationship. This has indicated that economic growth has been a crucial factor in Thailand. These outcomes have depicted that Thailand should concern about the rules implemented that impact growth [36].

Fig 7 visualises the causality between CO_2 emissions and REC consumption in a global context. In the short term (Scale 0-16) the years 1998,1999,2004,2011 and 2015 to 2019 depict a bidirectional relationship between CO_2 emissions and consumption of REC at high and medium frequencies. In 1996 there was a mixed relationship among the two variables with no causality.

Fig 7. Wavelet coherence: CO_2 vs REC for World

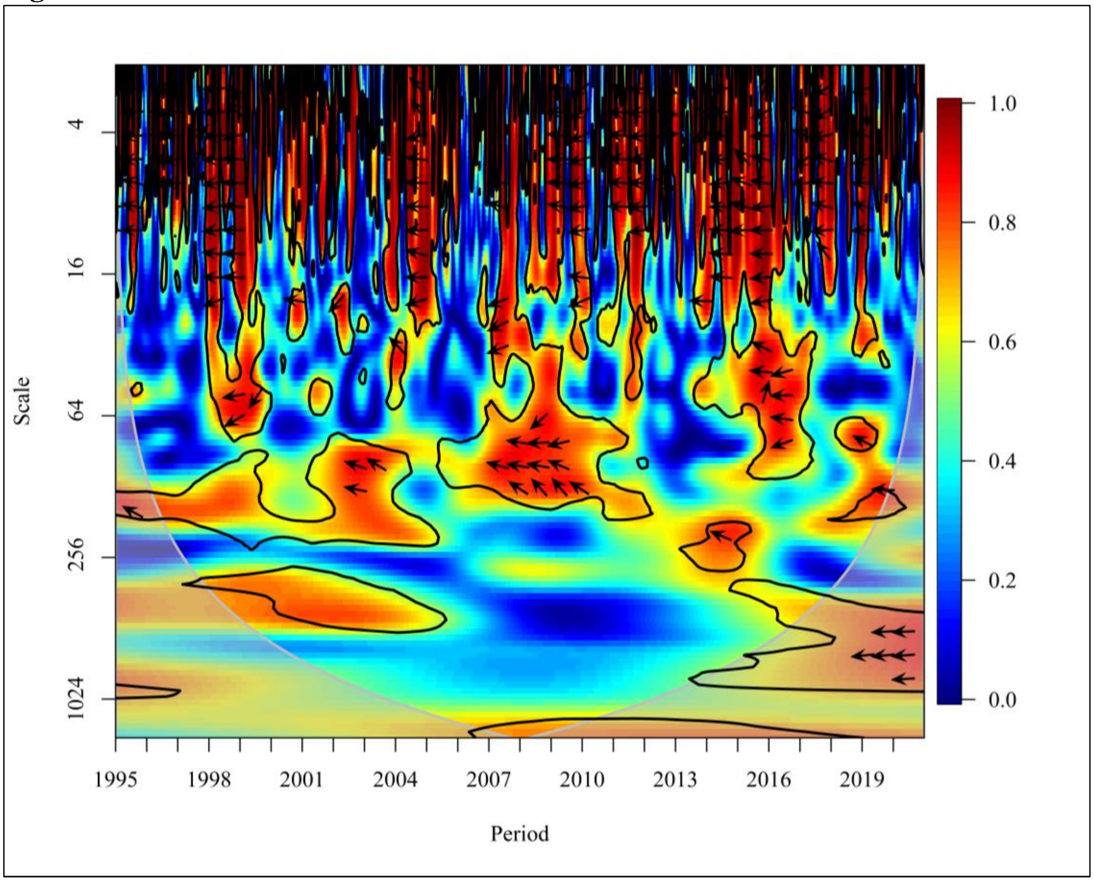

A study conducted to determine the bidirectional causality between energy consumption and carbon emission explains that Ghana's fuel energy consumption has resulted in CO_2 emissions. Therefore, the energy penetration has not reached the level required to start the reduction of CO_2 emissions as confirmed by a study [37]. Further, the years 1995 and 2007 depict that consumption of REC caused a reduction in CO_2 emission while year's 2010. Exceptionally, 2012 depicts that CO_2 emission causes a negative impact on the consumption of

REC at high and medium frequencies. In the medium term, globally (Scale 16-256), a bidirectional causality is depicted in the years 2002, 2007 to 2010, and 2015 to 2016 at high frequencies. Further, unidirectional causality is depicted in the years 2001, 2004, 2014, and 2018 in which REC has caused a negative impact on CO_2 emission. The years 1998 and 1999 show a unidirectional relationship where CO_2 negatively impacts REC. In the long term (Scale 256 – 1024) shows low frequency with no relationship between the variables in a global context. In contrast, a global study conducted based on 138 countries supports a bidirectional energy emission nexus between REC and CO_2 Emission. Research shows REC increases carbon emissions, while capital market investment boosts REC [63].

Unlike in the global context, Fig 8 shows high-income country category has a mixed relationship between CO_2 and REC in the short term (Scale 0-16) at high and medium frequencies. A bidirectional causality is depicted in most years while a unidirectional causality is depicted in 1999 when CO_2 emissions caused negatively on REC at 5% significance.

Fig 8. Wavelet coherence: CO_2 vs REC for High-income countries

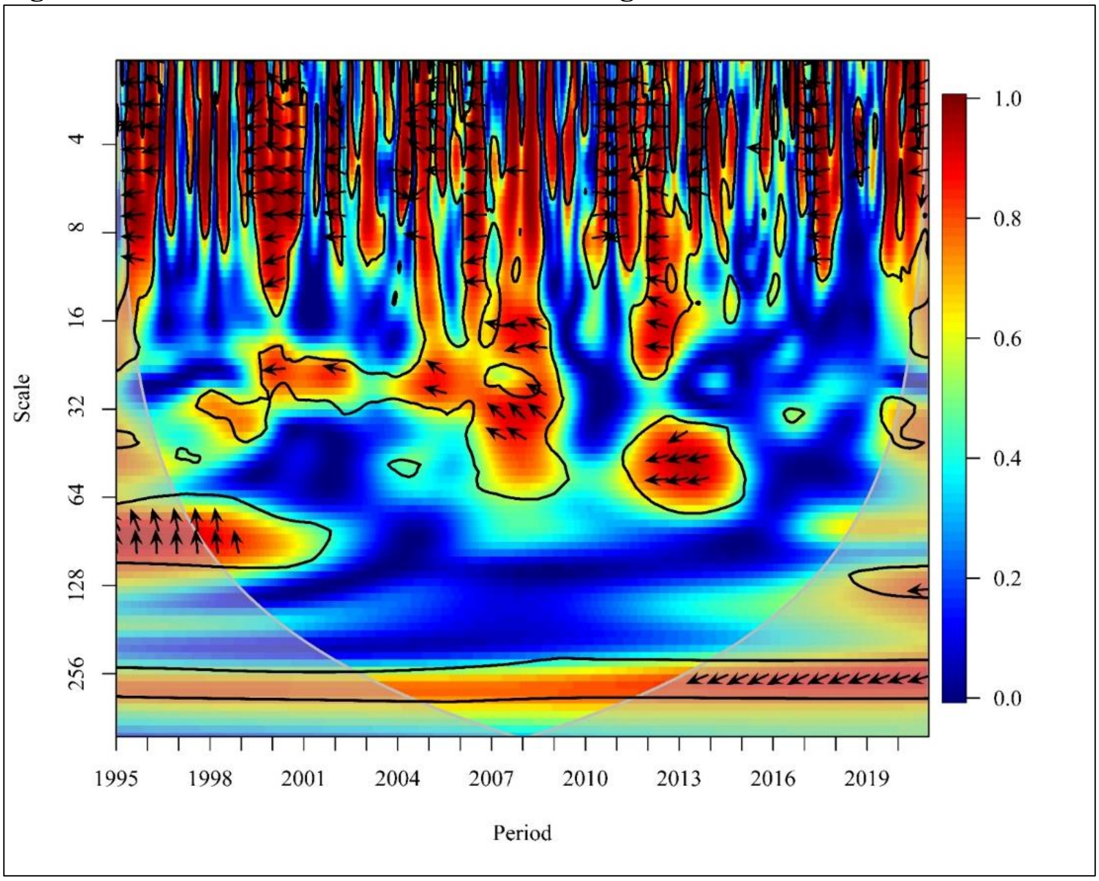

Stepping away from normality, towards the latter part of 2016, REC has positively caused CO_2 emissions. In the medium term (Scale 16 – 64), a bidirectional causality is depicted in the latter half of 2007 till the end of 2008 and 2012 at high frequency while the unidirectional relationship is shown in 2002, and 2005 at high and medium frequency with 5% significance. In the long term (Scale 64 – 256) unlike in the global context which did not show any relationship, the high-income category depicts a unidirectional relationship in the years 1997 and 1998. In relation to the study conducted in Japan reveals wavelet coherence for CO_2 and REC. Between 1993 and 1997, the series was out of sync with CO_2 emissions [64]. Also, 2009 to 2010 the measures act in phase with CO_2 and REC which aligns with the findings of the study. Overall, the findings reveal that higher usage of REC lowers CO_2 emissions. Thus, investment in RE is environmentally beneficial.

In Fig 9, for the low-income category, in the short term (Scale 0-8) the beginning of 1999 shows a bidirectional relationship while at the end of the year and 2004 a unidirectional relationship is shown which REC causes CO_2 emission. 2000 and 2007 years show a unidirectional relationship in which CO_2 negatively impacts REC with high frequency at 5% significance.

Fig 9. Wavelet coherence: CO_2 vs REC for Low-income countries

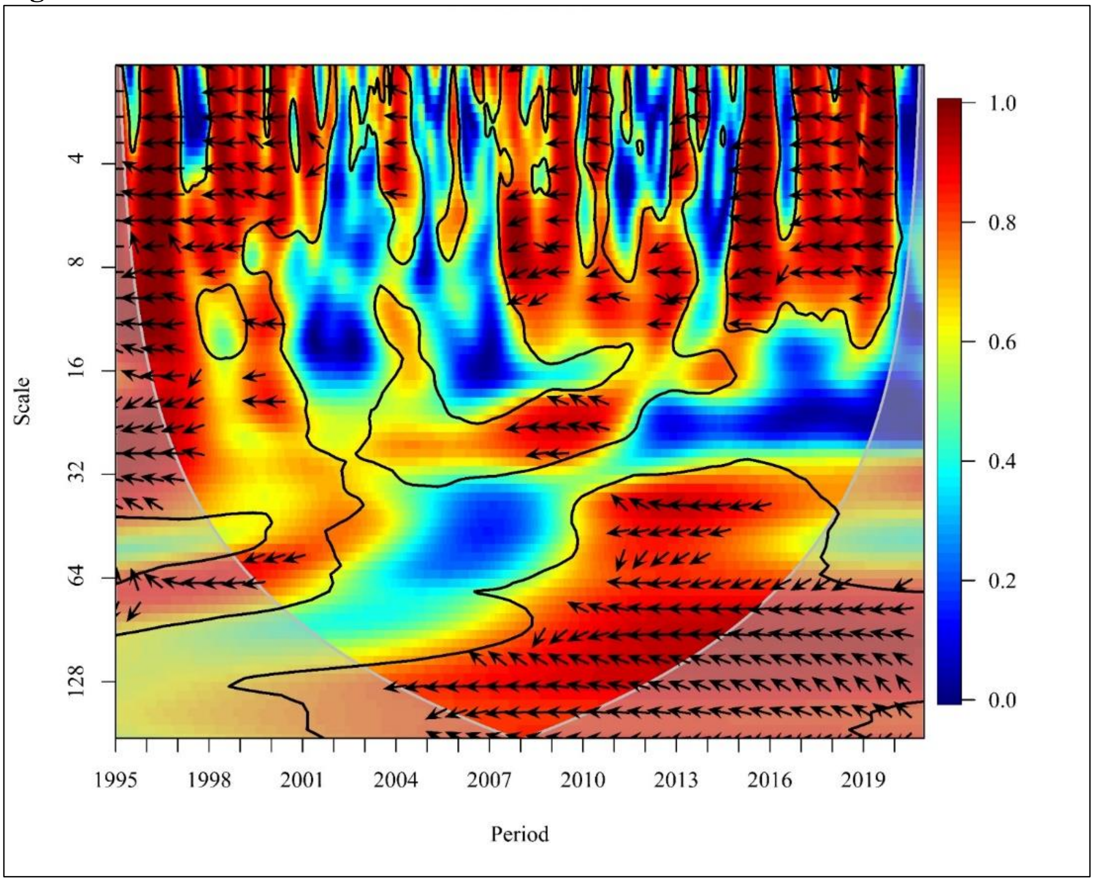

The year 2008 shows a different behaviour of the variable which is that CO_2 has caused a positive impact t to REC at high frequency. Considering the medium term (Scale 8-32) which shows a negative relationship between CO_2 and REC at high and medium frequency, a bidirectional causality is visible from 1996 to 2000 and in 2009 and 2010. Further, a unidirectional relationship is depicted in 2011 where REC caused CO_2, and years 2007 to 2008, CO_2 has led to REC negatively at a 5% significance level. Exceptional behaviour is visible

where a unidirectional positive causality from CO_2 to REC is depicted in the years 2012 and 2015. High and medium frequencies are depicted in the years 1998 to 2017 with a negative relationship whereas a bidirectional causality is shown from 2006 to 2015 in the long term (Scale 32 – 128) at 5% significance. Unlike in the global scale or high-income country category, the low-income country category shows a unidirectional relationship in 1999, 2000, 2004, and 2005 where CO_2 causes REC at a high frequency. Furthermore, a clear bidirectional causality between CO_2 and REC is visible from 2006 to 2015.

Fig 10 visualises the causality in lower-middle-income countries from 1995 to 2020 in the short term (Scale 0 – 16) as a bidirectional relationship between CO_2 and REC which is commonly shown throughout the period, except for 1997, 2001, 2009, and 2012 to 2016 which visualises unidirectional relationships at high frequency with 5% significance.

Fig 10. Wavelet coherence: CO_2 vs REC for Lower middle-income countries

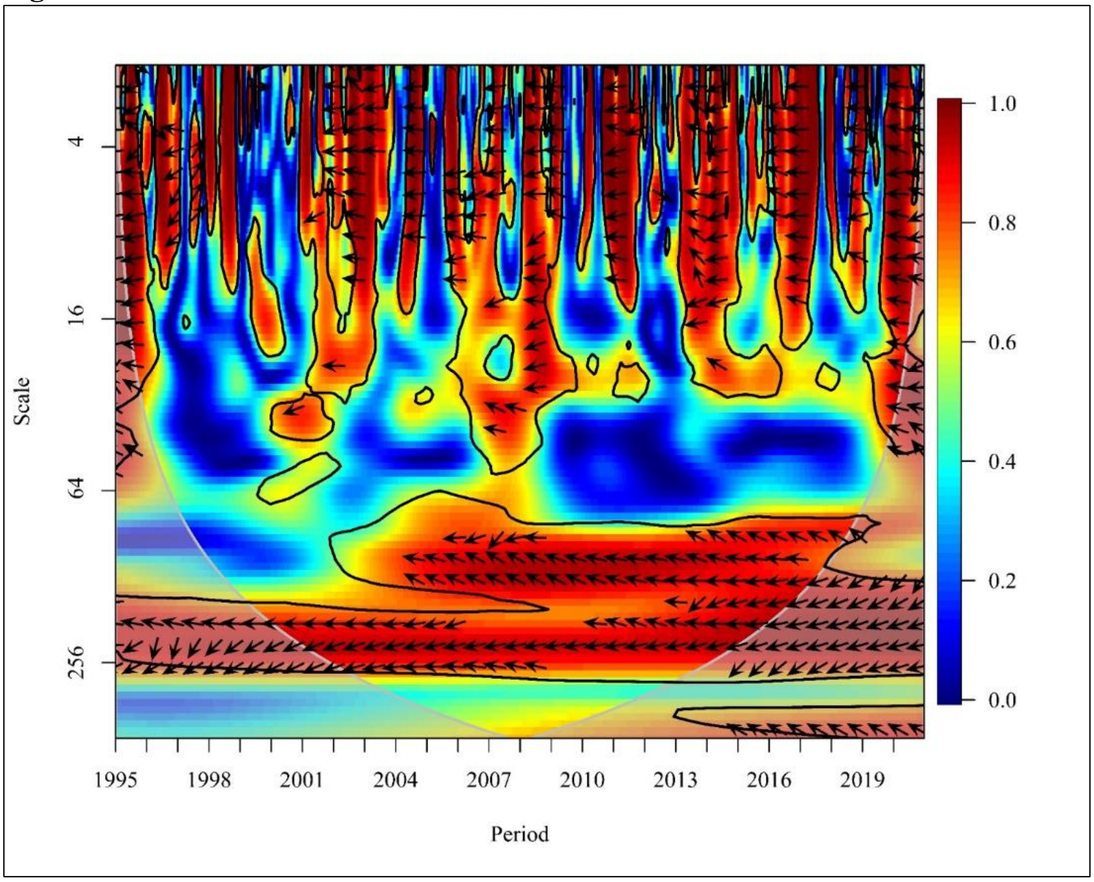

The year 2001 shows a one-way causality in which CO_2 impacts REC negatively while in 2009 and 2016 REC causes CO_2 negatively. Further, in 1997 there was a unidirectional positive causality in which REC caused CO_2 and in 2012 CO_2 positively caused REC at 5% significance. In the medium term (Scale 16 – 64), only the low middle-income category exhibits no bidirectional causation when compared to the medium term of other country categories. Unidirectional causality is visible in the years 2007, 2014, and 2020 where REC causes negative to CO_2. Years 2000, 2008, and 2013 show a unidirectional relationship where CO_2 negatively causes REC at high frequency with 5% significance. In the long term (Scale 64 – 256) from 2000 to 2018, there is a negative relationship between the CO_2 and REC where the years 2004 to 2018 specifically show a bidirectional causality between the two variables at a high and medium frequency at 5% significance. Further, a unidirectional relationship is indicated where CO_2 causes negative REC from 2000 to 2003.

In Fig 11, the upper middle-income country category, in the short term (Scale 0-16), bidirectional relationship is depicted in 1995, 1999 to 2004, 2007 to 2008, 2013 to 2014, 2016, and 2019 at a high and low frequency at 5% significance level.

Fig 11. Wavelet coherence: CO_2 vs REC for Upper middle-income countries

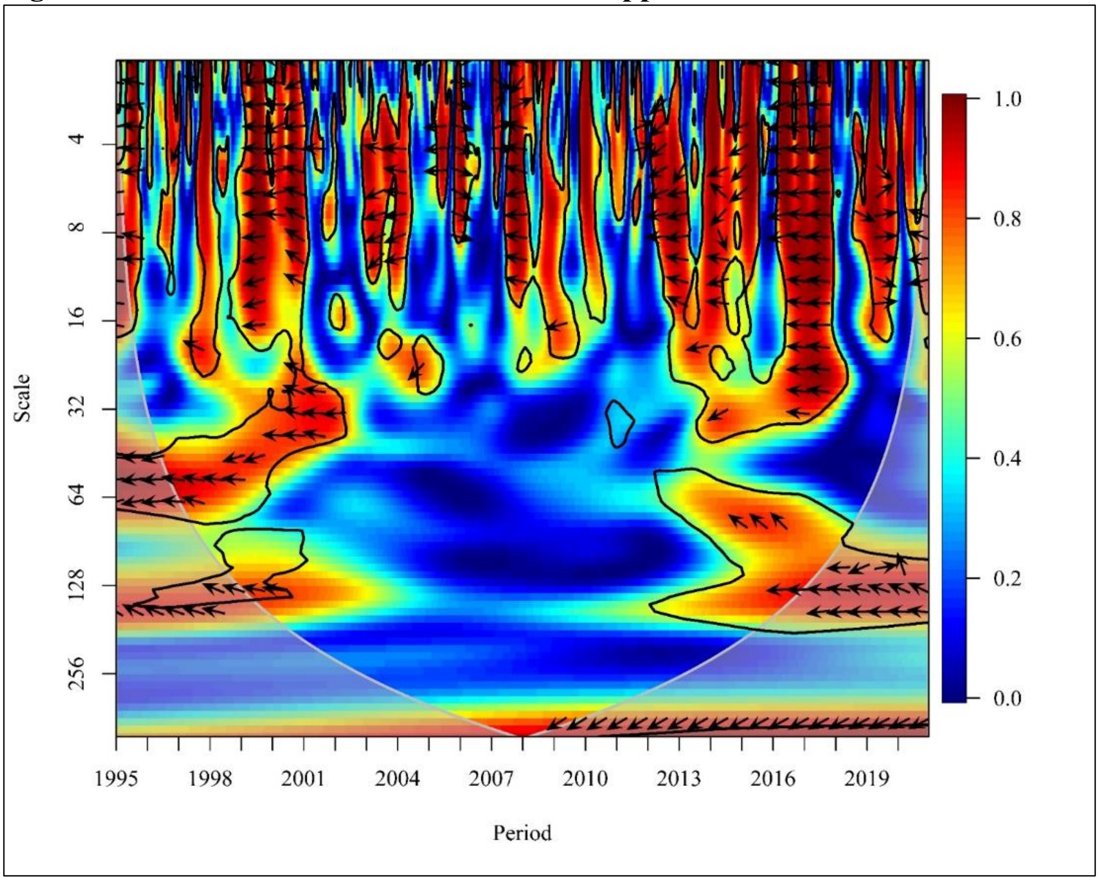

In the years 1996, 2005, 2012 and 2015 there is a unidirectional relationship where CO_2 impacts negatively to REC. Examining the positive correlation further, we find that, in 2006 and 2018, CO_2 positively caused REC, while in 2011, REC positively caused CO_2. In the middle term (Scale 16-64) in upper middle-income countries, bidirectional relationships are depicted in 1997 to 2001 at high frequency, while unidirectional relationships are visible in 2004, 2009, and 2014 where CO_2 has caused REC. Further, in the years 1997 and 2017, consumption of REC has negatively impacted CO_2 emissions. In the long term (Scale 64-256), there is a negative relationship from 1998 to 2001 and 2013 to 2018 in high and medium frequency. The years 1998 to 2001 and 2014 to 2016 depict a unidirectional relationship where REC has caused CO_2 emission negatively at a 5% significance level. A study on the impact of REC, PGDP, and net exports on consumer-based CO_2 emissions shows that Dynamic Ordinary

Least Square's estimation of REC lowers CO_2 emissions over time with an increase of 1% in REC and a decrease of 0.26% in CO_2 emissions. On the other hand, empirical results show that PGDP and imports have a beneficial impact on CO_2 emissions, which is a 1% rise in PGDP increasing by 0.46% of CO_2 [65].

The Usage of non-renewable energy like coal, natural gases, and petroleum has increased significantly over the last four decades in the period from 1980 to 2016 [66]. In the global context, a bidirectional causality was depicted negatively in 1995, 1998 to 1999, 2005, and from 2016 to 2019 with high frequency at 5% significance as shown in Fig 12. An empirical study which was done using data observed from 65 countries revealed a bidirectional causality in the short and long term. The causation for the results of a bidirectional causality is due to the industrial and economic growth in the world [39]. In the years 2007, 2009, and 2015, a unidirectional causality was depicted where CO_2 causes NREC. In 2010, NREC became the major cause of CO_2 emission. The variations of NREC and CO_2 in the medium term (16 -64) in the years 1997 and 2018 show a positive relationship and a bidirectional causality was identified from 2002 to 2004, 2007 to 2009, and 2016 to 2017.

Fig 12. Wavelet coherence: CO_2 vs NREC for World

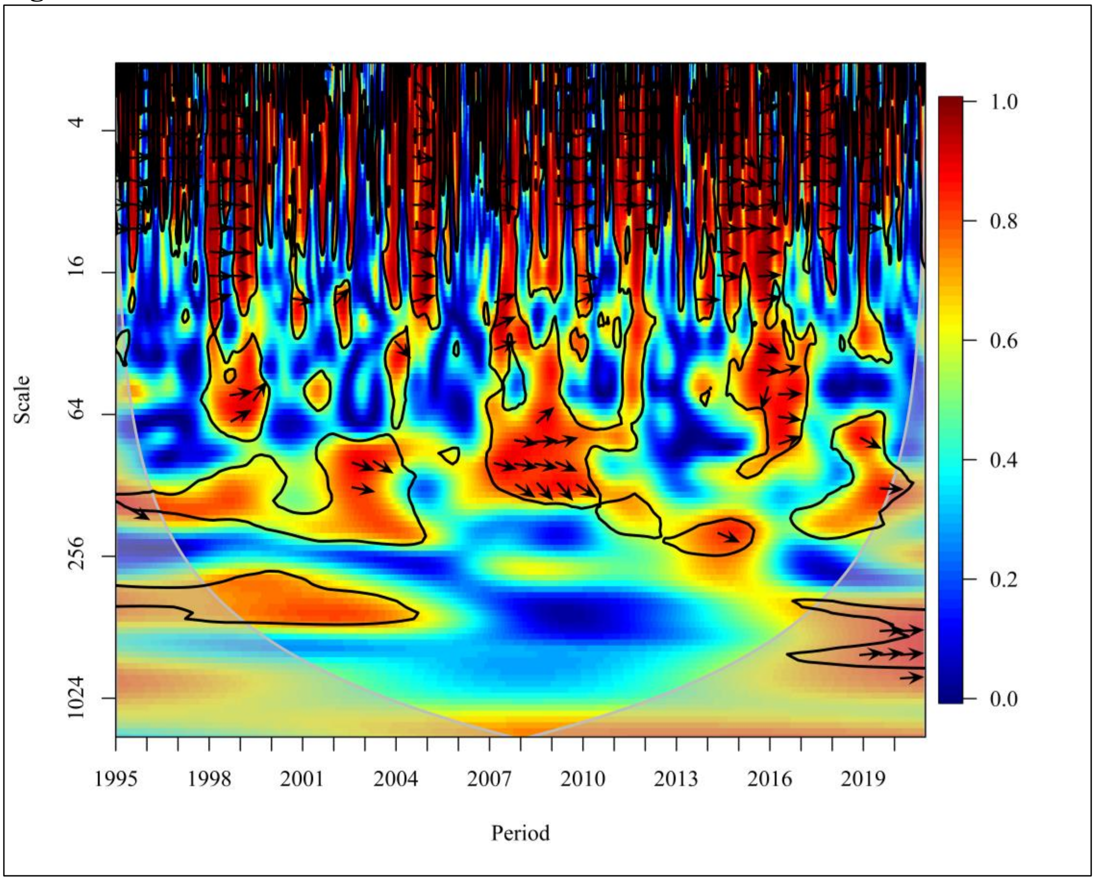

In the years 1998 to 1999, a unidirectional causality was identified where NREC led to CO_2 emission at a 5% significance level. A comparative study done in Thailand and BRICS countries has supported the result that an increase in consumption of non-renewable energy has led to an increase in CO_2 emission [50]. Furthermore, a unidirectional causality is depicted in 2001, 2014, and 2019 where CO_2 has caused positively on NREC with high and medium frequencies at a 5% significance level.

Fig 13 depicts, in high-income countries, a short term (Scale 0-16) and bidirectional causality between CO_2 emission and NREC for the period except for 1999 and the 1st half of 2004 which shows a unidirectional relationship where NREC has a positive cause to CO_2 emissions at 5% significance level. CO_2 emission has shown a negative and significant result which depicts that increases in CO_2 emission have contributed to a reduction in the use of non-

renewable energy in the short run [67]. In the medium term (Scale from 16- 64), a positive relationship at high significance is seen from 2000 to 2008 and 2012 to 2013.

Fig 13. Wavelet coherence: CO_2 vs NREC for High-income countries

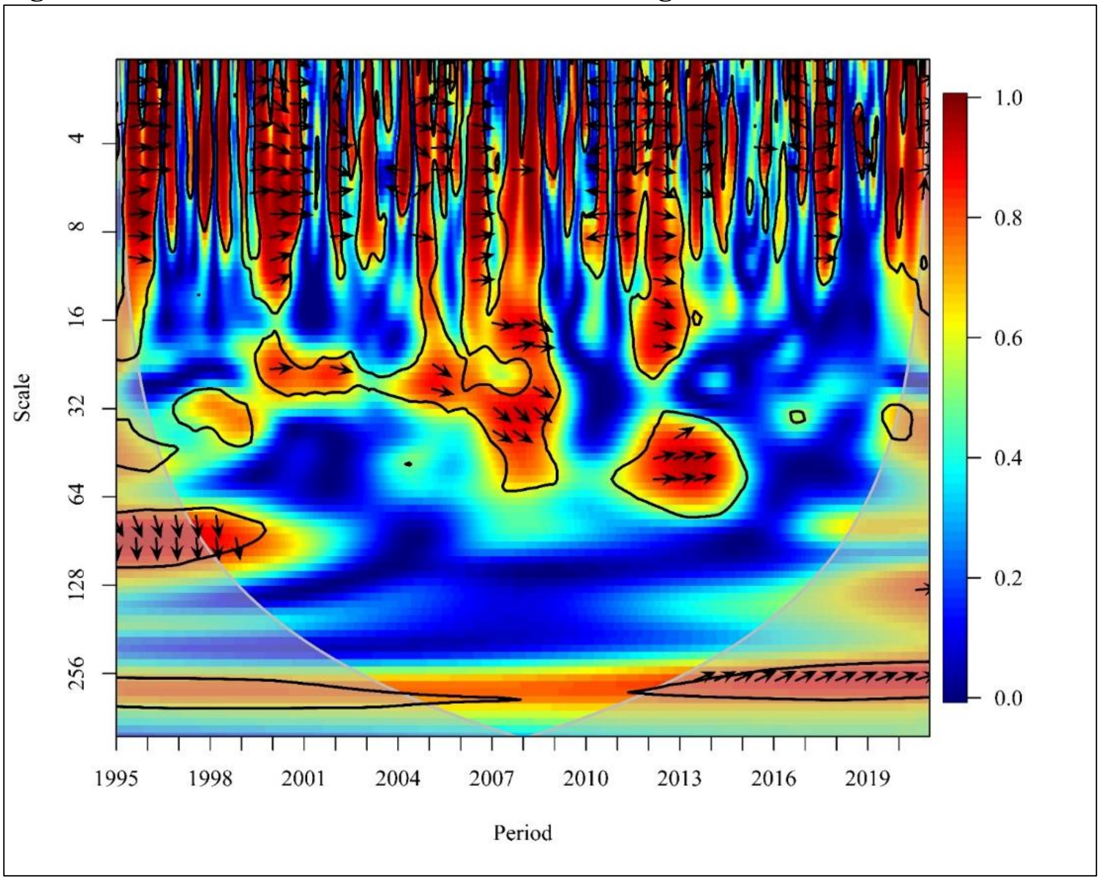

A bidirectional causality is visible from 2007 to 2008 and 2012 to 2013. Unidirectional relationships are visible in 2000 where NREC led to CO_2 emission while in the years 2002 and 2005, CO_2 led to an increase in NREC. Considering the long term (Scale 64 – 257) from 1998 to 1999, a unidirectional relationship is visible where CO_2 has led to an increase in NREC. The rest of the period shows low frequency.

In low-income countries from 1995 to 2019, a positive relationship is shown between CO_2 and NREC with mixed frequencies in the short term (Scale 0 – 8) which is illustrated in

Fig 14. In the years 1999, 2001, 2015, and from 2017 to 2019, a bidirectional relationship is depicted at high frequencies. Unidirectional relationships where NREC has led to CO_2 emission are visible in the years 1998, 2000, 2008 to 2009, and 2012 to 2013.

Fig 14. Wavelet coherence: CO_2 vs NREC for Low-income countries

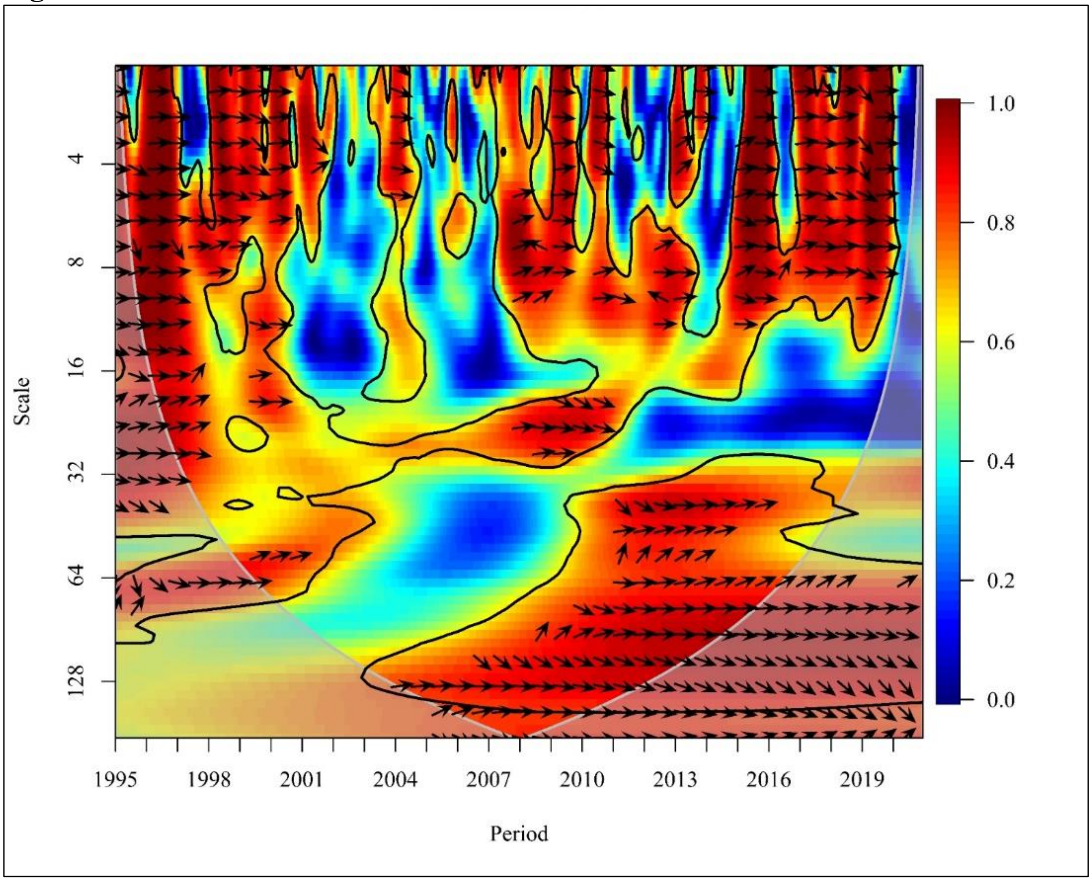

The years 1995 to 1997, 2004, and 2010 depict unidirectional causality where CO_2 had led to an increase in NREC at high frequency at a 5% significance level. Results of medium term (Scale from 8 – 32) from 1997 to 2000 and from 2009 to 2010 show a bidirectional causality with high frequency. NREC has led to CO_2 emissions showing a unidirectional causality in 2006, 2008 and 2013 while CO_2 has led to an increase in NREC in 2004 to 2011, and 2015. In the long term (Scale 32-128) from 2008 to 2013, a bidirectional causality is shown while in 1999 to 2000 and 2014 to 2016, a unidirectional causality is depicted where NREC leads to increase CO_2. Also, from 2006 to 2007, CO_2 has led positively to NREC.

In Fig 15, the Lower middle-income countries in the short-run (0 – 16) show a bidirectional causality in the years 1996,1997, from 2002 to 2006, 2008, 2011, and 2013 at a high frequency. There is a bidirectional causality among CO_2 emission and NREC with a 95% confidence level [25]. In 2013, there was a significant hike in NREC-led CO2 emissions compared to 2004 and 2011.

Fig 15. Wavelet coherence: CO_2 vs NREC for Lower middle-income countries

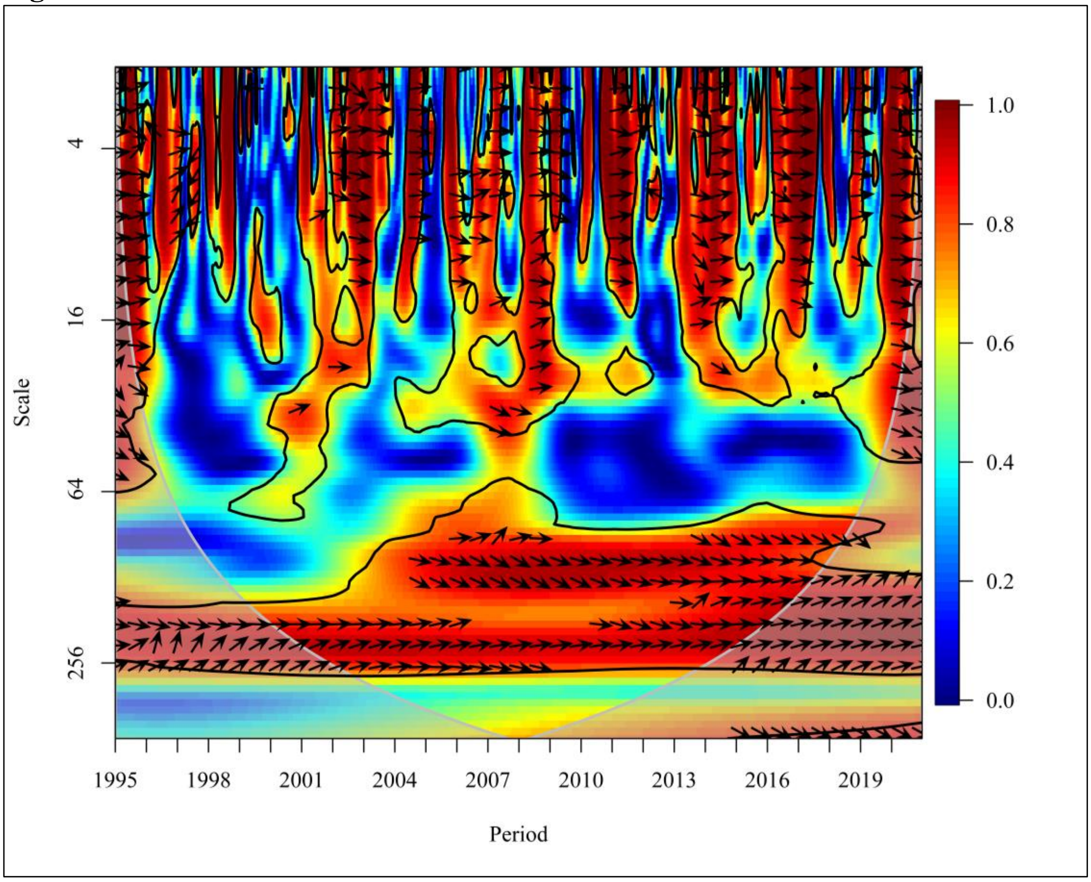

In the long term (64 – 256), a bidirectional causality has been identified from 2004 to 2016. A unidirectional relationship in which NREC Causes CO_2 is visible in 1995, 1997, 2001, 2007, and 2014. CO_2 has caused positive effects on NREC in the years 1997, 2009, and from 2015 to 2016. Exceptionally in the years 1996 and 2012, a negative relationship where NREC led to a decrease in CO_2 emission is depicted. In long term (Scale 64 – 256) shows bidirectional causality between NREC and CO_2 at high frequency from 2004 to 2016. It is proven by the

evidence generated by the results that there is bidirectional causality between CO_2 emission and NREC [39]. From 2001 to 2003, there has been a unidirectional causality where NREC positively caused CO_2.

In the upper middle-income countries years 1995,2001, 2003 to 2004, 2007 to 2008, 2013 to 2014, and 2019 in short-term (0 -16) resulted in a bidirectional causality between CO_2 emission and NREC at high frequency in Fig 16. 1997, 1999 to 2000, 2005, and 2015 to 2016 show right downward arrows revealing NREC has affected positively CO_2 emission at a 5% significance level.

Fig 16. Wavelet coherence: CO_2 vs NREC for Upper middle-income countries

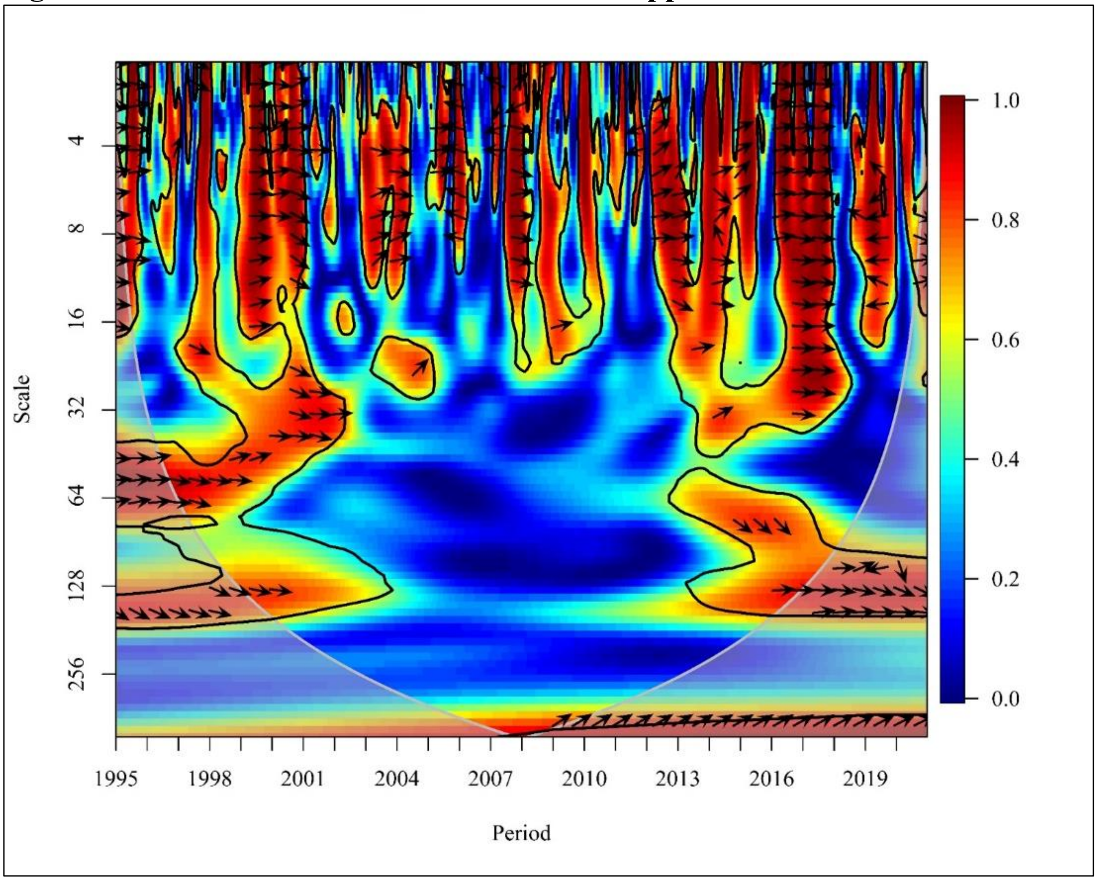

In exception to normal behaviour, 2005, 2011, and 2012 a unidirectional negative causality is shown at high frequency. In the medium term (Scale 16 – 64) from 1997 to 1999 show a bidirectional relationship. The future unidirectional relationship of NREC causing CO_2

is depicted in 2004, 2009, and 2013 to 2014. Also, CO_2 positively led to NREC from 2001 to 2002 and 2017 at a 5% significance level. In the long run (64 – 256) the results show the right downward arrow in 1998 and 2015 to 2016 with high and low frequencies revealing that CO_2 emission has caused NREC. A similar study reveals that there is a unidirectional causality running from CO_2 to NREC in EU countries [67].

In this study, the causality will be analysed for three periods: short-term, medium-term, and long-term from 1995 to 2020 with different frequencies. A summarisation of the correlation and causality of the variables for the aforementioned time period classified by five years is shown in Fig 17,

Fig 17. Summary of Wavelet Coherence Graph

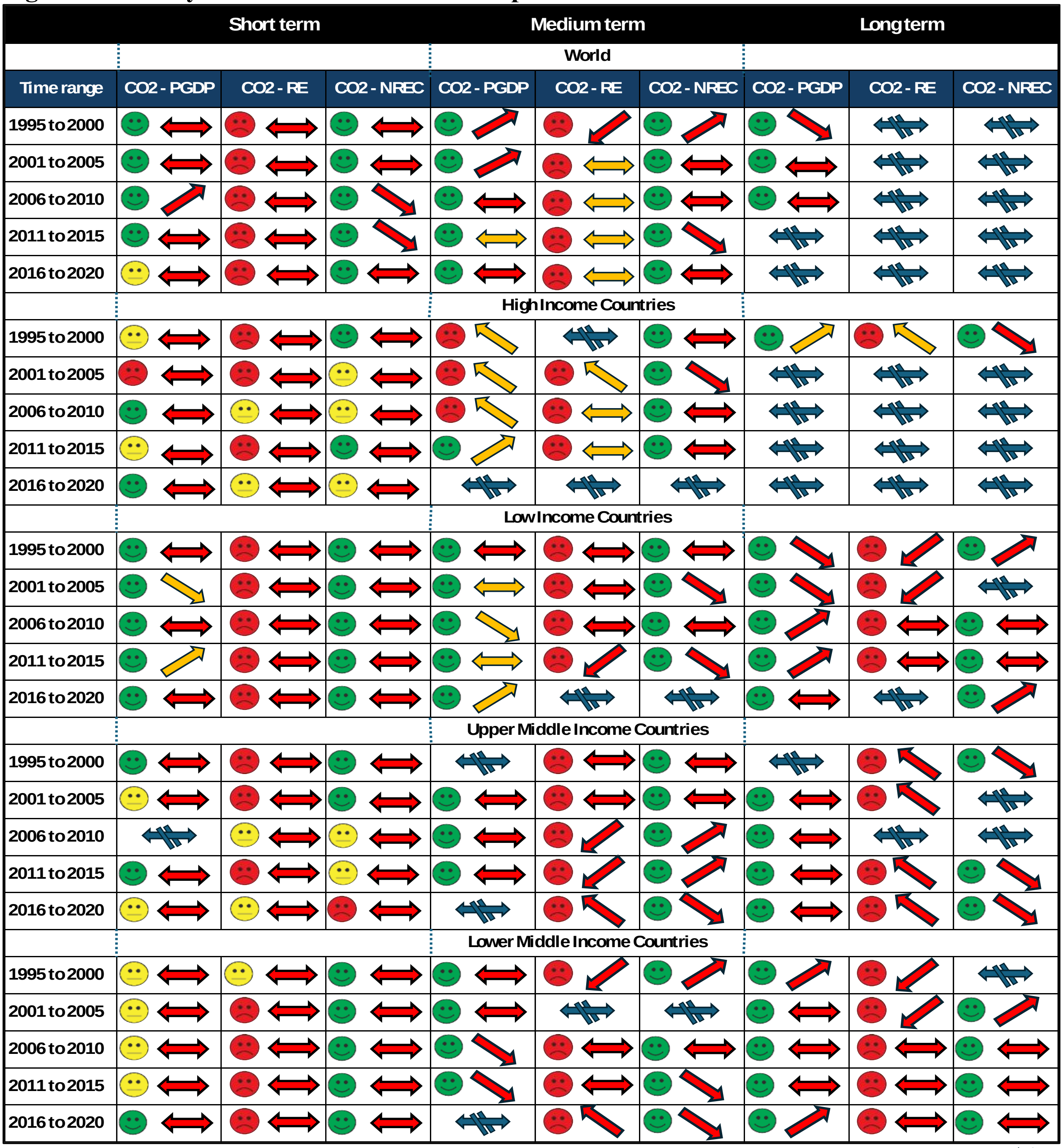

Note – Second variable causes the first variable, First Variable causes second variable. Positive, negative, and mixed impacts respectively, red colour arrow depicts high frequency and yellow colour arrow depicts the high and medium frequency., No Causality

Source: Authors' Compilation.

The results of the wavelet coherence indicate the existence of a significant correlation between PGDP and CO_2 emission and energy consumption and CO_2 emission in all the time scales. In

short term, medium term and long term, PGDP has both bidirectional relationship and unidirectional relationships in a global context which is similar to the study based on 30 countries which has shown that the countries that depend on fossil fuels have had economic growth while increasing the environmental deterioration like CO_2 emission with a mixed relationship while centering for bi-directional causality [68]. But in short term there is a one-way causality between PGDP and CO2 emission except for the 2006 to 2010 time range.

Further, all the country categories except for high income and low middle income have a unidirectional causality. Focusing on medium term, high income shows a one-way causality throughout the time period. In the long term, all the categories have one-way causality except for the upper middle-income category. REC also has a mixed causality with both bi-directional relationships and unidirectional relationships. In short, there is a negative relationship with bidirectional causality throughout the time range [69, 70] and in contrast, [71] it shows no causality globally and region-wise. In the medium term, there is a one-way causality from 2006 to 2020 in upper-middle-income and lower-middle-income categories in the first and the last five years of the given time period.

In the long term, there is no causality shown globally. One causality can be seen in the first period of high income and throughout the upper middle income with no causality from 2006 to 2010. Further, a mixed causality can be seen in lower income and lower middle-income categories. Similarly, NREC also has both bidirectional and unidirectional relationships. In the short term, there is a bidirectional relationship globally except for the years 2006 to 2015. In the medium term similar to REC there is a one-way causality in upper middle income, and it shows a right upward behaviour which explains NREC causes CO_2 in upper middle income from 2006 to 2015 and a downward arrow, where CO2 causes NREC in lower middle income from 2011 to 2020. Moreover, in comparison to the REC directional behaviour there is a large

number of one-way causalities. Further, in the long term, there is a one-way causality throughout in upper middle income.

The Granger causality test results obtained for each country under each income category are shown in S2 Appendix, S3 Appendix, S4 Appendix. Before the Granger causality test, the unit root test was applied to ascertain the stationarity of the variables. Considering the relationship between CO_2 emission and PGDP, RE, and NRE in general there is a reasonable confirmation that Granger causes each other in all income categories.

Table 54: Summary findings of Wavelet Coherence and Granger causality between CO_2 and PGDP, CO_2 and RE, and CO_2 and NRE

Income Category	Wavelet Coherence	Granger Causality
Global	$\leftrightarrow$	$\leftrightarrow$
High Income	$\leftrightarrow$	$\leftrightarrow$
Low Income	$\leftrightarrow$	$\leftrightarrow$
Upper Middle Income	$\leftrightarrow$	$\leftrightarrow$
Lower Middle Income	$\leftrightarrow$	$\leftrightarrow$

Source: Authors' Compilation.

Cross-country results of Granger causality between CO_2 and PGDP are presented in S2 Appendix. The results show that under the high-income country category, the countries Finland, Poland, Romania, and the Slovak Republic show clear bidirectional causalities between CO_2 and PGDP. Furthermore, 19 other countries show unidirectional behaviour where 13 countries show CO_2 causing GDP and 6 other countries show GDP causing CO_2 with the rest of the 22 countries showing no causality. Considering the upper middle-income country category, the Dominican Republic shows two-way causality while Libya, Albania, Dominica Marshall Islands, and St. Vincent depict unidirectional causality of CO_2 causing PGDP, and Grenadines and Botswana, El Salvador, Gabon, Jamaica, Malaysia, Mexico depict PGDP causing CO_2. A study reveals a bidirectional relationship between CO_2 and GDP in economies in transition, a unidirectional relationship for developing countries, and no causality for

developed and least developed countries, covering 152 countries, analysed through Granger causality [59].

Another study reveals that GDP growth drives CO_2 emission in the US, France, Australia, and Germany with a unidirectional relationship and growth in CO_2 emission drives that in GDP in China, India, Brazil and Japan [72].

Additionally, low-middle-income countries and low-income countries show unidirectional and no causality among the countries. In the low middle-income category Algeria, Angola, Benin, Haiti, Kiribati, Lesotho, Mongolia, Samoa, Tajikistan, Tunisia, and Bangladesh show that CO_2 causes GDP while Cabo Verde, Ghana, Guinea, Honduras, Nepal, Vanuatu, Bhutan, and all other 33 countries show no causality. Under the low-income country category, Togo, the Central African Republic, Chad, Niger, and the Syrian Arab Republic show unidirectional relationships with CO_2 causality to PGDP while Madagascar, Mali, Sudan, and Uganda show GDP Causality to CO_2 also all other 11 countries show no causality.

Considering all country categories, the cross-country Granger results of CO_2 and RE are shown in S3 Appendix. Bidirectional causalities are present in Qatar, Trinidad and Tobago, France, Portugal, Romania, and Uruguay in the High-income country category. Furthermore, unidirectional relationships with CO_2 causality to RE are depicted in Seychelles, Australia, Cyprus, Finland, Germany, Israel, and New Zealand while RE causality to CO_2 is depicted in Andorra, Canada, Iceland, Panama, Singapore, Spain, St. Kitts, and Nevis, Switzerland, and Saudi Arabia. 23 other countries show no causality. Upper middle-income country categories St. Lucia, Equatorial Guinea, and Maldives show bidirectional Granger while 11 other countries show unidirectional causality. Out of the 11 countries Belize, Guatemala, Mauritius, Jamaica, Turkey, and South Africa show CO_2 causes RE, while Cuba, Fiji, Costa Rica, Indonesia, and Kazakhstan show GDP causing CO_2.

Guinea shows a bidirectional relationship under the low-middle-income country category, with Honduras, India, and Tunisia showing unidirectional causality of CO_2 causing RE. In addition, Algeria, Cabo Verde, Egypt, Arab Rep., Mongolia, and Bolivia countries show that RE causes CO_2 emission. 42 countries show no causality under low-middle-income countries. Considering Low-income countries, the Granger between CO_2 and RE is bidirectional in the Central African Republic, Chad, and Congo, Dem. Rep. Unidirectional Granger with CO_2 causing RE is present in Mali, Rwanda, and Uganda while RE causing CO_2 is present in Guinea-Bissau, Niger, Sudan, and the Syrian Arab Republic. A study done to analyse the causal relationship between the same variables in the current study reveals a strong relationship among variables signifying that there is a bidirectional relationship between RE and CO_2 [43].

Similar results to the Granger causality between CO_2 emission and RE are depicted cross-country for CO_2 emission and NRE, which are presented in S4 Appendix.

Conclusion and policy implications

The causality among economic growth, renewable energy, and non-renewable energy has been analysed using the wavelet coherence method for the period 1998-2020. Throughout the period in short term a bidirectional causality has been shown between CO_2 emission among renewable and non-renewable energy in most years while no causality has resulted from 2006-2010. In medium term, from 2016-2020, in high-income countries, from 1995 to 2000 and 2016 to 2020 in low-income countries, in the long term no causality among CO_2 emission and NREC has been shown from 1995 – 2020 and in high income countries from 2001-2020. Negative bi-directional causality was found in the world from 1995-2020 and in low-income countries in the short term while a positive relationship has resulted from 1995 – 2020 in low-income countries. Further to confirm the results taken by Wavelet coherence, cross-country analysis

has been done using the Granger causality test which reveals mixed relationships for different countries which includes, bi-direction, uni-direction and no causality between CO_2 and PGDP, CO_2 and REC and CO_2 and NREC. Further comparing the two methodologies reveals all the income group categories have a bidirectional causality between all three variables mentioned above in general.

Policymakers can concentrate on initiatives like promoting renewable energy sources, directing investments towards sustainable technologies, strengthening and enforcing regulations to reduce emissions. The utilisation of renewable energy from hydro, solar, wind, biomass, and geothermal sources presents a more sustainable option, leading to reduced CO_2 emissions [73, 74]. Comprehending the scale and features of these CO_2 emissions is crucial for devising effective mitigating strategies [75-77].

Achieving net zero carbon emissions typically involves two approaches. Firstly, it involves offsetting carbon emitted into the atmosphere by investing in sustainable technologies like carbon capture and storage. Secondly, it entails averting future carbon emissions by prioritising renewable energy sources. Various organisations have launched collaborative projects to curb CO_2 emissions from industries such as cement and steel, which are recognised as major contributors to CO_2 emissions in production economies. Many studies have shown that switching to renewable energy leads to lower CO_2 emissions. With widespread concern about greenhouse gases, it's expected that the level of CO_2 emissions will greatly affect how much renewable energy has been used [78-84]. Governments should take actions by implementing programs such as promoting public transportation for urban traveling and imposing additional charges for the use of personal vehicles within cities.

By effectively addressing the intricate relationship between economic growth, renewable energy and non-renewable energy, ~~there's a lot of~~our study highlights several

policy~~ies~~ recommendations~~ed to emphasise~~supported by the results ~~taken through~~of the granger causality test and wavelet coherence approach. Promoting renewable energy through incentives such as grants, tax incentives, low interest loans to mitigate carbon emissions which fosters the ~~development of the economy~~economic development. Another crucial strategy is investing in sustainable technologies, including~~.~~ c~~C~~arbon capturing technologies, energy efficient systems, green hydrogen technologies and smart grids. ~~have been a huge impact to mitigate carbon emissions which is also have shown a significant impact to economic growth.~~ Moreover, the causality results indicate, even though economic expansions tend to increase carbon emissions, with the implementation of these innovative technologies can mitigate this~~hat~~ relationship. Enforcing regulatory frameworks is also a pivotal policy implication which aligns with the causality of the variables in the study. By e~~E~~mploying stringent emissions regulations and carbon pricing mechanisms like carbon taxes, ensures that polluters internalise the costs of pollution, thereby incentivising cleaner technologies.~~pricing mechanisms such as carbon taxes ensure that the cost of pollution is internaliszed by emitters . This gives the encouragement to innovate cleaner technologies.~~ Addressing urban transportation emissions is critical,~~One major problem for carbon emission is urban transport . There are many suggested policies for urban transportation which drive towards sustainability. S~~with strategies such as electric vehicles, non-motorized transportation modes will tend to reduce emissions. Improving the urban transportation infrastructure will enhance economic efficiency while~~and~~ reducing~~e~~ pollution. Additionally, international collaborations will ~~be also~~play a crucial role ~~which is a global implication~~in addressing ~~to~~ global climate change challenges. Collaborative efforts such as technological transfer agreements, fostering partnerships, participating in~~on of~~ global climate initiatives, and developing policy frameworks, ~~national polices has~~have created synergies that amplify the positive impact~~ways~~ of individual national efforts in mitigating CO_2 emissions.

However, it is important to acknowledge the limitations of this study, including the unavailability of data which are relevant for the recent years. Although wavelet analysis is an effective method for analysing non-stationary signals in time-frequency space, its interpretation of correlation patterns can be distorted due to its sensitivity to wavelet basis and parameter selection. A limited number of studies have been done using the wavelet coherence method in a global context. Future researchers could incorporate additional variables to provide a more comprehensive understanding of emissions. In addition, indicators like other greenhouse gas emissions can be used for future studies as possible extensions.

References

1. Acaroğlu H, Güllü M. Climate change caused by renewable and non-renewable energy consumption and economic growth: A time series ARDL analysis for Turkey. Renewable Energy. 2022;193:434-47. doi: https://doi.org/10.1016/j.renene.2022.04.138.
2. Box-Steffensmeier JM, Burgess J, Corbetta M, Crawford K, Duflo E, Fogarty L, et al. The future of human behaviour research. Nature Human Behaviour. 2022;6(1):15-24. Epub 2022/01/29. doi: https://doi.org/10.1038/s41562-021-01275-6. PubMed PMID: 35087189.
3. Fetisov V, Gonopolsky AM, Davardoost H, Ghanbari AR, Mohammadi AH. Regulation and impact of VOC and CO2 emissions on low-carbon energy systems resilient to climate change: A case study on an environmental issue in the oil and gas industry. Energy Science & Engineering. 2023;11(4):1516-35. doi: https://doi.org/10.1002/ese3.1383.
4. Hao Y. Effect of Economic Indicators, Renewable Energy Consumption and Human Development on Climate Change: An Empirical Analysis Based on Panel Data of Selected Countries. Frontiers in Energy Research. 2022;10. doi: https://doi.org/10.3389/fenrg.2022.841497.
5. Ozdemir AC. Decomposition and decoupling analysis of carbon dioxide emissions in electricity generation by primary fossil fuels in Turkey. Energy. 2023;273. doi: https://doi.org/10.1016/j.energy.2023.127264.
6. Wu Y, Zhu Q, Zhu B. Decoupling analysis of world economic growth and CO2 emissions: A study comparing developed and developing countries. Journal of Cleaner Production. 2018;190:94-103. doi: https://doi.org/10.1016/j.jclepro.2018.04.139.
7. World Bank. The World Bank Annual Report 2018. 2018.
8. Holechek JL, Geli HME, Sawalhah MN, Valdez R. A Global Assessment: Can Renewable Energy Replace Fossil Fuels by 2050? Sustainability. 2022;14(8). doi: https://doi.org/10.3390/su14084792.
9. Al-mulali U, Weng-Wai C, Sheau-Ting L, Mohammed AH. Investigating the environmental Kuznets curve (EKC) hypothesis by utilizing the ecological footprint as an indicator of environmental degradation. Ecological Indicators. 2015;48:315-23. doi: https://doi.org/10.1016/j.ecolind.2014.08.029.

10. Destek MA, Sarkodie SA. Investigation of environmental Kuznets curve for ecological footprint: The role of energy and financial development. Science of the Total Environment. 2019;650(Pt 2):2483-9. Epub 2018/10/08. doi: https://doi.org/10.1016/j.scitotenv.2018.10.017. PubMed PMID: 30293003.
11. Adebayo TS, Beton Kalmaz D. Determinants of CO2 emissions: empirical evidence from Egypt. Environmental and Ecological Statistics. 2021;28(2):239-62. doi: https://doi.org/10.1007/s10651-020-00482-0.
12. Bakhsh K, Rose S, Ali MF, Ahmad N, Shahbaz M. Economic growth, CO(2) emissions, renewable waste and FDI relation in Pakistan: New evidences from 3SLS. Journal of Environmental Management. 2017;196:627-32. Epub 2017/04/02. doi: https://doi.org/10.1016/j.jenvman.2017.03.029. PubMed PMID: 28364712.
13. Liu X, Bae J. Urbanization and industrialization impact of CO2 emissions in China. Journal of Cleaner Production. 2018;172:178-86. doi: https://doi.org/10.1016/j.jclepro.2017.10.156.
14. Akbota A, Baek J. The Environmental Consequences of Growth: Empirical Evidence from the Republic of Kazakhstan. Economies. 2018;6(1). doi: https://doi.org/10.3390/economies6010019.
15. Ahmed Z, Wang Z, Ali S. Investigating the non-linear relationship between urbanization and CO2 emissions: An empirical analysis. Air Quality, Atmosphere & Health. 2019;12(8):945-53. doi: https://doi.org/10.1007/s11869-019-00711-x.
16. Kirikkaleli D, Kalmaz DB. Testing the moderating role of urbanization on the environmental Kuznets curve: empirical evidence from an emerging market. Environ Sci Pollut Res Int. 2020;27(30):38169-80. Epub 2020/07/04. doi: https://doi.org/10.1007/s11356-020-09870-2. PubMed PMID: 32617821.
17. Odugbesan JA, Adebayo TS. The symmetrical and asymmetrical effects of foreign direct investment and financial development on carbon emission: evidence from Nigeria. SN Applied Sciences. 2020;2(12). doi: https://doi.org/10.1007/s42452-020-03817-5.
18. Chen X, Rahaman MA, Murshed M, Mahmood H, Hossain MA. Causality analysis of the impacts of petroleum use, economic growth, and technological innovation on carbon emissions in Bangladesh. Energy. 2023;267. doi: https://doi.org/10.1016/j.energy.2022.126565.
19. Mohamed Yusoff NY, Ridzuan AR, Soseco T, Wahjoedi, Narmaditya BS, Ann LC. Comprehensive Outlook on Macroeconomic Determinants for Renewable Energy in Malaysia. Sustainability. 2023;15(5):3891. PubMed PMID: doi:10.3390/su15053891.
20. IIEA. World Energy Investment Outlook 2023. 2023.
21. DW Planet A. Which will be the engine of the future? Youtube2023.
22. Financial Times. Can hydrogen help the world reach net zero? Youtube2023.
23. IIEA. World Energy Investment Outlook 2022. 2022.
24. Kabir M, Habiba U, Iqbal MZ, Shafiq M, Farooqi ZR, Shah A, et al. Impacts of anthropogenic activities & climate change resulting from increasing concentration of Carbon dioxide on environment in 21st Century; A Critical Review. IOP Conference Series: Earth and Environmental Science. 2023;1194(1). doi: https://doi.org/10.1088/1755-1315/1194/1/012010.
25. Awodumi OB, Adewuyi AO. The role of non-renewable energy consumption in economic growth and carbon emission: Evidence from oil producing economies in Africa. Energy Strategy Reviews. 2020;27. doi: https://doi.org/10.1016/j.esr.2019.100434.
26. Wang S, Li G, Fang C. Urbanization, economic growth, energy consumption, and CO2 emissions: Empirical evidence from countries with different income levels. Renewable and Sustainable Energy Reviews. 2018;81:2144-59. doi: https://doi.org/10.1016/j.rser.2017.06.025.

27. Le TH. Connectedness between nonrenewable and renewable energy consumption, economic growth and CO2 emission in Vietnam: New evidence from a wavelet analysis. Renewable Energy. 2022;195:442-54. doi: https://doi.org/10.1016/j.renene.2022.05.083.
28. Islami FS, Prasetyanto PK, Kurniasari F. The Effect of Population, GDP, Non-Renewable Energy Consumption and Renewable Energy Consumption on Carbon Dioxide Emissions in G-20 Member Countries. International Journal of Energy Economics and Policy. 2022;12(2):103-10. doi: https://doi.org/10.32479/ijeep.12548.
29. AlNemer HA, Hkiri B, Tissaoui K. Dynamic impact of renewable and non-renewable energy consumption on CO2 emission and economic growth in Saudi Arabia: Fresh evidence from wavelet coherence analysis. Renewable Energy. 2023;209:340-56. doi: https://doi.org/10.1016/j.renene.2023.03.084.
30. Fatima T, Shahzad U, Cui L. Renewable and nonrenewable energy consumption, trade and CO2 emissions in high emitter countries: does the income level matter? Journal of Environmental Planning and Management. 2020;64(7):1227-51. doi: https://doi.org/10.1080/09640568.2020.1816532.
31. Khalfaoui R, Tiwari AK, Khalid U, Shahbaz M. Nexus between carbon dioxide emissions and economic growth in G7 countries: fresh insights via wavelet coherence analysis. Journal of Environmental Planning and Management. 2021;66(1):31-66. doi: https://doi.org/10.1080/09640568.2021.1978062.
32. Toumi S, Toumi H. Asymmetric causality among renewable energy consumption, CO(2) emissions, and economic growth in KSA: evidence from a non-linear ARDL model. Environmental Science and Pollution Research. 2019;26(16):16145-56. Epub 2019/04/12. doi: https://doi.org/10.1007/s11356-019-04955-z. PubMed PMID: 30972668.
33. Chekouri SM, Chibi A, Benbouziane M. Economic growth, carbon dioxide emissions and energy consumption in Algeria: a wavelet coherence approach. World Journal of Science, Technology and Sustainable Development. 2021;18(2):172-89. doi: https://doi.org/10.1108/wjstsd-12-2020-0097.
34. Acheampong AO. Economic growth, CO2 emissions and energy consumption: What causes what and where? Energy Economics. 2018;74:677-92. doi: https://doi.org/10.1016/j.eneco.2018.07.022.
35. Ardakani MK, Seyedaliakbar SM. Impact of energy consumption and economic growth on CO2 emission using multivariate regression. Energy Strategy Reviews. 2019;26. doi: https://doi.org/10.1016/j.esr.2019.100428.
36. Adebayo TS, Akinsola GD. Investigating the Causal Linkage Among Economic Growth, Energy Consumption and CO2 Emissions in Thailand: An Application of the Wavelet Coherence Approach. International Journal of Renewable Energy Development. 2021;10(1):17-26. doi: https://doi.org/10.14710/ijred.2021.32233.
37. Appiah MO. Investigating the multivariate Granger causality between energy consumption, economic growth and CO 2 emissions in Ghana. Energy Policy. 2018;112:198-208. doi: https://doi.org/10.1016/j.enpol.2017.10.017.
38. Aswadi K, Jamal A, Syahnur S, Nasir M. Renewable and Non-renewable Energy Consumption in Indonesia: Does it Matter for Economic Growth? International Journal of Energy Economics and Policy. 2023;13(2):107-16. doi: https://doi.org/10.32479/ijeep.13900.
39. Ben Jebli M, Kahia M. The interdependence between CO2 emissions, economic growth, renewable and non-renewable energies, and service development: evidence from 65 countries. Climatic Change. 2020;162(2):193-212. doi: https://doi.org/10.1007/s10584-020-02773-8.

40. P. Goupillaud AGaJM. Cycle octave and related transfors in seismic signal. Journal of Applied Geophysics. 1984;23(1):85-102. doi: https://doi.org/10.1016/0016-7142(84)90025-5.
41. Acquaah YT, Gokaraju B, Tesiero RC, Monty GH. Thermal Imagery Feature Extraction Techniques and the Effects on Machine Learning Models for Smart HVAC Efficiency in Building Energy. Remote Sensing. 2021;13(19). doi: https://doi.org/10.3390/rs13193847.
42. Rhif M, Ben Abbes A, Farah I, Martínez B, Sang Y. Wavelet Transform Application for/in Non-Stationary Time-Series Analysis: A Review. Applied Sciences. 2019;9(7). doi: https://doi.org/10.3390/app9071345.
43. Attiaoui I, Toumi H, Ammouri B, Gargouri I. Causality links among renewable energy consumption, CO2 emissions, and economic growth in Africa: evidence from a panel ARDL-PMG approach. Environmental Science and Pollution Research. 2017;24(14):13036-48. doi: 10.1007/s11356-017-8850-7.
44. Peng H, Tan X, Li Y, Hu L. Economic Growth, Foreign Direct Investment and CO2 Emissions in China: A Panel Granger Causality Analysis. Sustainability. 2016;8(3):233. PubMed PMID: doi:10.3390/su8030233.
45. Dharmapriya N, Edirisinghe S, Gunawardena V, Methmini D, Jayathilaka R, Dharmasena T, et al. Towards a greener future: examining carbon emission dynamics in Asia amid gross domestic product, energy consumption, and trade openness. Environmental Science and Pollution Research. 2024;31(14):21488-508. doi: 10.1007/s11356-024-32475-y.
46. Raihan A, Voumik LC, Ridwan M, Ridzuan AR, Jaaffar AH, Yusoff NYM. From growth to green: Navigating the complexities of economic development, energy sources, health spending, and carbon emissions in Malaysia. Energy Reports. 2023;10:4318-31. doi: https://doi.org/10.1016/j.egyr.2023.10.084.
47. Banday UJ, Aneja R. Renewable and non-renewable energy consumption, economic growth and carbon emission in BRICS. International Journal of Energy Sector Management. 2020;14(1):248-60. doi: 10.1108/IJESM-02-2019-0007.
48. Attanayake K, Wickramage I, Samarasinghe U, Ranmini Y, Ehalapitiya S, Jayathilaka R, et al. Renewable energy as a solution to climate change: Insights from a comprehensive study across nations. PLOS ONE. 2024;19(6):e0299807. doi: 10.1371/journal.pone.0299807.
49. Ponce P, Khan SAR. A causal link between renewable energy, energy efficiency, property rights, and CO2 emissions in developed countries: A road map for environmental sustainability. Environmental Science and Pollution Research. 2021;28(28):37804-17. doi: 10.1007/s11356-021-12465-0.
50. Phatchapa Boontome AT, Jaruwan Chontanawat. Investigating the causal relationship between non-renewable and renewable energy consumption, CO2 emissions and economic growth in Thailand. Energy Procedia. 2017;138:925-30. doi: https://doi.org/10.1016/j.egypro.2017.10.141.
51. Dogan E, Seker F. Determinants of CO2 emissions in the European Union: The role of renewable and non-renewable energy. Renewable Energy. 2016;94:429-39. doi: https://doi.org/10.1016/j.renene.2016.03.078.
52. Dogan E, Seker F. The influence of real output, renewable and non-renewable energy, trade and financial development on carbon emissions in the top renewable energy countries. Renewable and Sustainable Energy Reviews. 2016;60:1074-85. doi: https://doi.org/10.1016/j.rser.2016.02.006.
53. Lakshani S, Sandaruwan C, Fernando C, Vidyapathirana G, Jayathilaka R, Munasinghe S. From short to long term: Dynamic analysis of FDI and net export in global regions. PLoS One. 2023;18(9):e0291301. Epub 2023/09/14. doi:

https://doi.org/10.1371/journal.pone.0291301. PubMed PMID: 37708132; PubMed Central PMCID: PMCPMC10501631.

54. Adebayo TS, Onyibor K, Akinsola GD. The impact of major macroeconomic variables on foreign direct investment in Nigeria: evidence from a wavelet coherence technique. SN Business & Economics. 2020;1(1). doi: https://doi.org/10.1007/s43546-020-00018-5.
55. Wijesekara C, Tittagalla C, Jayathilaka A, Ilukpotha U, Jayathilaka R, Jayasinghe P. Tourism and economic growth: A global study on Granger causality and wavelet coherence. PLoS One. 2022;17(9):e0274386. Epub 2022/09/13. doi: https://doi.org/10.1371/journal.pone.0274386. PubMed PMID: 36094926; PubMed Central PMCID: PMCPMC9467361.
56. Granger CWJ. Investigating Causal Relations by Econometric Models and Cross-spectral Methods. Econometrica. 1969;37(3):424-38. doi: https://doi.org/10.2307/1912791.
57. Jayawardhana T, Jayathilaka R, Nimnadi T, Anuththara S, Karadanaarachchi R, Galappaththi K, et al. The cost of aging: Economic growth perspectives for Europe. PLoS One. 2023;18(6):1-20. doi: https://doi.org/10.1371/journal.pone.0287207.
58. Galappaththi K, Jayathilaka R, Rajamanthri L, Jayawardhana T, Anuththara S, Nimnadi T, et al. Economy and elderly population, complementary or contradictory: A cross-continental wavelet coherence and cross-country Granger causality study. PLoS One. 2023;18(1):e0278716. Epub 2023/01/27. doi: https://doi.org/10.1371/journal.pone.0278716. PubMed PMID: 36701291; PubMed Central PMCID: PMCPMC9879505.
59. Dissanayake H, Perera N, Abeykoon S, Samson D, Jayathilaka R, Jayasinghe M, et al. Nexus between carbon emissions, energy consumption, and economic growth: Evidence from global economies. PLoS One. 2023;18(6):e0287579. Epub 2023/06/23. doi: https://doi.org/10.1371/journal.pone.0287579. PubMed PMID: 37352276; PubMed Central PMCID: PMCPMC10289335.
60. Munir Q, Lean HH, Smyth R. CO2 emissions, energy consumption and economic growth in the ASEAN-5 countries: A cross-sectional dependence approach. Energy Economics. 2020;85. doi: https://doi.org/10.1016/j.eneco.2019.104571.
61. Torun E, Akdeniz ADA, Demireli E, Grima S. Long-Term US Economic Growth and the Carbon Dioxide Emissions Nexus: A Wavelet-Based Approach. Sustainability. 2022;14(17). doi: https://doi.org/10.3390/su141710566.
62. Abban OJ, Hongxing Y, Nuta AC, Dankyi AB, Ofori C, Cobbinah J. Renewable energy, economic growth, and CO2 emissions contained Co-movement in African oil-producing countries: A wavelet based analysis. Energy Strategy Reviews. 2022;44. doi: https://doi.org/10.1016/j.esr.2022.100977.
63. Ofori-Sasu D, Abor JY, Agyekum Donkor GN, Otchere I. Renewable energy consumption and carbon emissions in developing countries: the role of capital markets. International Journal of Sustainable Energy. 2023;42(1):1407-29. doi: https://doi.org/10.1080/14786451.2023.2268857.
64. Adebayo TS, Kirikkaleli D. Impact of renewable energy consumption, globalization, and technological innovation on environmental degradation in Japan: application of wavelet tools. Environment, Development and Sustainability. 2021;23(11):16057-82. doi: https://doi.org/10.1007/s10668-021-01322-2.
65. Mukhtarov S, Aliyev F, Aliyev J, Ajayi R. Renewable Energy Consumption and Carbon Emissions: Evidence from an Oil-Rich Economy. Sustainability. 2022;15(1). doi: https://doi.org/10.3390/su15010134.
66. Khan H, Khan I, Binh TT. The heterogeneity of renewable energy consumption, carbon emission and financial development in the globe: A panel quantile regression approach. Energy Reports. 2020;6:859-67. doi: https://doi.org/10.1016/j.egyr.2020.04.002.

67. Shafiei S, Salim RA. Non-renewable and renewable energy consumption and CO2 emissions in OECD countries: A comparative analysis. Energy Policy. 2014;66:547-56. doi: https://doi.org/10.1016/j.enpol.2013.10.064.
68. Al-mulali U. Investigating the impact of nuclear energy consumption on GDP growth and CO 2 emission: A panel data analysis. Progress in Nuclear Energy. 2014;73:172-8. doi: https://doi.org/10.1016/j.pnucene.2014.02.002.
69. Saidi K, Ben Mbarek M. Nuclear energy, renewable energy, CO 2 emissions, and economic growth for nine developed countries: Evidence from panel Granger causality tests. Progress in Nuclear Energy. 2016;88:364-74. doi: https://doi.org/10.1016/j.pnucene.2016.01.018.
70. Amin N, Song H. The role of renewable, non-renewable energy consumption, trade, economic growth, and urbanization in achieving carbon neutrality: A comparative study for South and East Asian countries. Environmental Science and Pollution Research. 2023;30(5):12798-812. doi: 10.1007/s11356-022-22973-2.
71. Wu Z, Hou G, Xin B. The Causality between Participation in GVCs, Renewable Energy Consumption and CO2 Emissions. Sustainability. 2020;12(3). doi: https://doi.org/10.3390/su12031237.
72. Mou W. A Granger Causality Analysis between the GDP and CO2 Emissions of Major Emitters and Implications for International Climate Governance. Chinese Journal of Urban and Environmental Studies. 2018;06(01):1850004. doi: https://doi.org/10.1142/S2345748118500045.
73. Thirunavukkarasu M, Sawle Y, Lala H. A comprehensive review on optimization of hybrid renewable energy systems using various optimization techniques. Renewable and Sustainable Energy Reviews. 2023;176. doi: https://doi.org/10.1016/j.rser.2023.113192.
74. Zheng J, Du J, Wang B, Klemeš JJ, Liao Q, Liang Y. A hybrid framework for forecasting power generation of multiple renewable energy sources. Renewable and Sustainable Energy Reviews. 2023;172. doi: https://doi.org/10.1016/j.rser.2022.113046.
75. Rahman A, Richards R, Dargusch P, Wadley D. Pathways to reduce Indonesia's dependence on oil and achieve longer-term decarbonization. Renewable Energy. 2023;202:1305-23. doi: https://doi.org/10.1016/j.renene.2022.11.051.
76. Sambodo MT, Yuliana CI, Hidayat S, Novandra R, Handoyo FW, Farandy AR, et al. Breaking barriers to low-carbon development in Indonesia: deployment of renewable energy. Heliyon. 2022;8(4):e09304. Epub 2022/05/07. doi: https://doi.org/10.1016/j.heliyon.2022.e09304. PubMed PMID: 35520621; PubMed Central PMCID: PMCPMC9061260.
77. Zhou H, Awosusi AA, Dagar V, Zhu G, Abbas S. Unleashing the asymmetric effect of natural resources abundance on carbon emissions in regional comprehensive economic partnership: What role do economic globalization and disaggregating energy play? Resources Policy. 2023;85. doi: https://doi.org/10.1016/j.resourpol.2023.103914.
78. Acheampong AO, Adams S, Boateng E. Do globalization and renewable energy contribute to carbon emissions mitigation in Sub-Saharan Africa? Science of the Total Environment. 2019;677:436-46. Epub 2019/05/07. doi: https://doi.org/10.1016/j.scitotenv.2019.04.353. PubMed PMID: 31059886.
79. Alola AA, Bekun FV, Sarkodie SA. Dynamic impact of trade policy, economic growth, fertility rate, renewable and non-renewable energy consumption on ecological footprint in Europe. Sci Total Environ. 2019;685:702-9. Epub 2019/06/17. doi: https://doi.org/10.1016/j.scitotenv.2019.05.139. PubMed PMID: 31203164.
80. Koc S, Bulus GC. Testing validity of the EKC hypothesis in South Korea: role of renewable energy and trade openness. Environmental Science and Pollution Research.

2020;27(23):29043-54. Epub 2020/05/20. doi: https://doi.org/10.1007/s11356-020-09172-7. PubMed PMID: 32424750.

81. Menegaki AN. Growth and renewable energy in Europe: A random effect model with evidence for neutrality hypothesis. Energy Economics. 2011;33(2):257-63. doi: https://doi.org/10.1016/j.eneco.2010.10.004.
82. Paramati SR, Ummalla M, Apergis N. The effect of foreign direct investment and stock market growth on clean energy use across a panel of emerging market economies. Energy Economics. 2016;56:29-41. doi: https://doi.org/10.1016/j.eneco.2016.02.008.
83. Sadorsky P. Renewable energy consumption and income in emerging economies. Energy Policy. 2009;37(10):4021-8. doi: https://doi.org/10.1016/j.enpol.2009.05.003.
84. Salim RA, Rafiq S. Why do some emerging economies proactively accelerate the adoption of renewable energy? Energy Economics. 2012;34(4):1051-7. doi: https://doi.org/10.1016/j.eneco.2011.08.015.

Supporting Information

S1 Appendix. Data File

S2 Appendix. Cross-country analysis between CO_2 and PGDP for all income categories using Granger Causality

S3 Appendix. Cross-country analysis between CO_2 and RE for all income categories using Granger Causality

S4 Appendix. Cross-country analysis between CO_2 and NRE for all income categories using Granger Causality
