# Supplementary material for: Understanding the interplay of GDP, renewable, and non-renewable energy on carbon emissions: Global wavelet coherence and Granger causality analysis
Source: PLoS One. 2024 Sep 19;19(9):e0308780. doi: 10.1371/journal.pone.0308780 (PMC11412634; doi:10.1371/journal.pone.0308780)
Supplement: Attachment — Submitted filename: Response to Reviewers.docx [file pone.0308780.s005.docx]

**Point-by-Point Response to the Editor’s Comments**

Dear Editor,

Greetings!

We extend our sincere appreciation for your invaluable comments and suggestions provided. Your insights have played a crucial role in the revision and enhancement of our work. We are genuinely grateful for the opportunity to submit the revised version of our manuscript, originally titled " Understanding the Interplay of GDP, Renewable, and Non-Renewable Energy on Carbon Emissions: Global Wavelet Coherence Analysis " to PLOS ONE, submitted on 31^st^ March 2024.

We have diligently incorporated the suggested improvements, and the revised document includes marked modifications. For your convenience, we have prepared a table detailing the changes, with line numbers corresponding to the revised manuscript that incorporates track changes. Your continued support and guidance are highly valued, and we look forward to any further insights you or the reviewers may offer.

Please find the reference table below, aligning with the line numbers in the revised manuscript containing without track changes.

| **Comments of the Editor** | **Comments of the Authors** |
| --- | --- |
| ***Journal Requirement 1*** |  |
| Please ensure that your manuscript meets PLOS ONE's style requirements, including those for file naming. | In response to the reviewer's comment, we have carefully reviewed PLOS ONE's style requirements and made necessary adjustments to our manuscript. Specifically, we have renamed the figures according to the journal's guidelines and ensured that each file is saved in an acceptable format as individual files. We believe these revisions align with the journal's requirements. Thank you for bringing this to our attention. |
| ***Journal Requirement 2*** |  |
| Please note that your Data Availability Statement is currently missing the direct link to access each database. If your manuscript is accepted for publication, you will be asked to provide these details on a very short timeline. We therefore suggest that you provide this information now, though we will not hold up the peer review process if you are unable. | Thank you for bringing this to our attention. We appreciate the importance of providing direct access to the data underlying our study. Below, we have included the direct links to access each database corresponding to the variables analysed in our manuscript:   1) CO2 emission in Metric tons Per Capita - <https://data.worldbank.org/indicator/EN.ATM.CO2E.PC> 2) Per capita GDP (Current US$) - <https://data.worldbank.org/indicator/NY.GDP.PCAP.CD> 3) % of total final energy consumption - <https://data.worldbank.org/indicator/EG.FEC.RNEW.ZS> 4) 100 - % of total final energy consumption - <https://data.worldbank.org/indicator/EG.FEC.RNEW.ZS> |
| ***Additional Editor Comment*** |  |
| The method is too simple. The authors need to demonstrate their deep understanding on wavelets, otherwise, results are not reliable,it is not enough to only use one tool to do analysis. More statistical analysis should be added | We appreciate the reviewer's insightful comments regarding the methodology employed in our study. To address concerns about the robustness of our analysis, we have made significant enhancements to our methodology, particularly focusing on the utilization of cross-country Granger causality.   1) Introduction of Cross-Country Granger Causality: We have incorporated cross-country Granger causality tests to deepen our analysis and demonstrate a more comprehensive understanding of the relationships between variables. Specifically, we have examined the causality between CO_2_ emissions and key variables such as per capita GDP (PGDP), renewable energy (RE), and non-renewable energy (NRE). Line numbers (102 to 104) has incorporated to briefly explain the analysis. 2) Methodology Enhancement: We have provided a detailed introduction to our methodology, highlighting the incorporation of cross-country Granger causality tests. This addition can be found in the relevant section (line numbers (232 to 247). 3) Results Integration: The results of the Granger causality tests have been seamlessly integrated into our analysis, enhancing the depth and reliability of our findings. These results are presented in detail from (line numbers 632 to 685). The results obtained from the analysis is presented in S2 Appendix, S3 Appendix, S4 Appendix. 4) Incorporation into Abstract and Conclusion: To ensure transparency and coherence, we have reflected the inclusion of cross-country Granger causality tests in both the Abstract (line numbers 42 to 43) and the Conclusion (line numbers 696 to 701).   Additionally, we have introduced Violin plots to visually represent the dispersion of the data for CO2 emissions, PGDP, RE, and NRE across different country categories (line numbers 269 to 284), further enriching the analysis.  These enhancements not only address the reviewer's concerns but also contribute significantly to the rigor and depth of our study. We believe these additions strengthen the reliability and validity of our results. Thank you for the opportunity to improve our manuscript based on your valuable feedback. |
